# Diversity and enzymatic activity of Polish beehive products microbiota, and characterization of a novel β-galactosidase from *Paenibacillus* sp. 8

**DOI:** 10.1038/s41598-025-02561-3

**Published:** 2025-05-21

**Authors:** Aleksandra Rosińska, Marta Wanarska, Katarzyna Kozłowska-Tylingo, Michał Jurkowski

**Affiliations:** 1 Department of Biotechnology and Microbiology, Faculty of Chemistry, Gdansk University of Technology, 80-233 Gdansk, Poland; 2 Department of Pharmaceutical Technology and Biochemistry, Faculty of Chemistry, Gdansk University of Technology, 80-233 Gdansk, Poland; 3 Department of Physical Chemistry, Faculty of Chemistry, Gdansk University of Technology, 80-233 Gdansk, Poland

**Keywords:** Beehive products, *Paenibacillus*, β-Galactosidase, Lactose hydrolysis, Lactulose hydrolysis, Biotechnology, Microbiology

## Abstract

Microbial enzymes are widely used in many industries, and beehive products are an abundant source of microorganisms. In this study, bacteria, yeasts, and filamentous fungi were isolated from Polish honey and bee bread samples and were investigated as enzyme producers. The best producers of glycoside hydrolases were *Paenibacillus* spp., whereas *Micrococcus* spp. showed high proteolytic and lipolytic activity. The β-galactosidase-encoding *bgaP* gene from *Paenibacillus* sp. 8 was cloned and expressed in *Escherichia coli*. BgaP, a hexameric protein with a molecular mass of 466 kDa, was optimally active at pH 6.6-7.0 and 40 °C, and maintained 18% of its maximum activity at 10 °C. β-Galactosidase was strongly inhibited by galactose, moderately by glucose, and slightly by fructose; therefore, it hydrolyzed lactulose much more efficiently than lactose. The efficiency of lactose digestion by BgaP was increased to almost 80% by the l-arabinose isomerase-catalyzed bioconversion of galactose to tagatose. These features make BgaP β-galactosidase a potential candidate for application in lactulose detection and in the production of health-promoting sweeteners. Furthermore, the cell-free extract of *Paenibacillus* sp. 8 hydrolyzed lactose in milk and synthesized galactooligosaccharides at 10 °C, indicating the presence of β-galactosidase other than BgaP in bacterial cells, justifying further research.

**Supplementary Information:**

The online version contains supplementary material available at 10.1038/s41598-025-02561-3.

## Introduction

Enzymes play essential roles in living organisms; thus, nature is a vast and abundant area for harnessing biocatalysts. In addition, they have the potential to be game changers for humanity when utilized in industries, such as food and beverages, detergents, animal feed, pharmaceuticals, biofuels, textiles, personal care, cosmetics, wastewater, and agriculture^1^. One of the most urgent and crucial problems is climate change, which must be addressed immediately. Enzymes seem to hold promise for combating this issue. For example, enzymatic reactions utilized in industrial processes can lower the energy footprint, reduce waste production, and decrease chemical consumption, which can lead to decreased greenhouse gas emissions^2^. It is worth noting that the industrial enzyme market is constantly growing, and the estimated revenue forecast in 2030 is valued at USD 11.42 billion with a compound annual growth rate (CAGR) of 6.4% from 2024 to 2030^3^. Interestingly, even during the COVID-19 pandemic, the enzyme market showed a tendency to grow and was not affected as in other industries. This non-standard scenario could be correlated with the rising demand for more sustainable processes in some of the aforementioned industrial areas^4^.

Enzymes used on an industrial scale are primarily derived from microorganisms, namely, approximately 60% fungi, 24% bacteria, and 4% yeasts, because they provide high activity, high yields, cost reduction, and work simplicity^1^. Products manufactured using glycoside hydrolases, whose key enzymes are cellulases and amylases, are primarily used in the food and beverage, animal feed, and pharmaceutical industries, mainly for polysaccharide saccharification. These enzymes account for the majority of the market, with a 48.34% share in 2023. The second and third places in the global enzyme market are proteases and lipases, respectively^3^. Proteases are produced by many microorganisms, but bacteria of the *Bacillus* genus are mostly commercially exploited for proteases, which are extensively used for purposes such as modification, flavor improvement, and shelf-life extension in the food and beverage industry. These include cheese production (κ-casein hydrolysis), infant formula preparation, and baking (gluten hydrolysis). Microbial proteases have also been used to clarify beer and fruit juice. Furthermore, this group of enzymes has been used in detergents and waste management as an eco-friendly alternative to hazardous chemicals in poultry waste degradation and is commonly employed in leather soaking, bating, and dehairing^5^.

Microbial lipases are widely used in the dairy industry to hydrolyze milk fat, boost cheese flavor, accelerate cheese ripening, and produce butter, cream, and yogurt. These biocatalysts are also used in the fat and oil industries, tea processing, and baking. It is worth mentioning that lipases are used in leather processing, as an additive to detergents and cosmetics, as well as for the synthesis of pharmaceuticals and biodiesel through esterification and transesterification^6^.

Among the extensive industrial applications of biocatalysts, the food and beverage segment dominate the market, accounting for the largest share of revenue at 21.05% in 2023^3^. β-Galactosidase (lactase) is a particularly important enzyme used in the food industry. This enzyme not only hydrolyzes lactose, making milk and other dairy products suitable for lactose-intolerant individuals, which is considered a conventional usage, but also exhibits transglycosylation activity used in the synthesis of galactooligosaccharides (GOS), which have been used as prebiotics in infant formulas to stimulate the growth of probiotic bacteria belonging to the *Bifidobacterium* and *Lactobacillus* genera^7,8^. Another, no less important, application of β-galactosidase is the processing of dairy industry by-products, whey, and whey permeate (deproteinized whey). The hydrolysis of lactose into the monosaccharides glucose and galactose makes these by-products sweeter and more digestible, so they can be used as additives in food, beverages, and animal feed^8^. Moreover, a β-galactosidase/glucose oxidase/peroxidase biosensor was tested for the determination of lactose in milk and dairy products^9^. The global β-galactosidase market was valued at USD 193.6 million in 2020 and was projected to grow at a CAGR of 5.1% from 2020 to 2027. The lactase market size was 505.9 tons in 2020 and is expected to reach 700.0 tons in 2027. Yeast-based enzymes accounted for more than 44% of the global revenue in 2019; however, bacteria-based β-galactosidase is being increasingly used for lactose hydrolysis in food and dietary supplement industries^10^.

Beehive products, such as honey, bee-collected pollen, bee bread (naturally fermented pollen), royal jelly, and propolis (bee glue), are rich sources of various microorganisms, including bacteria, yeasts, and filamentous fungi. Moreover, each individual bee product is a different micro-niche associated with distinct microbial community. These microbial communities are formed as combinations of honey bee (*Apis mellifera*) microbiota and microorganisms originating from the environment inside and outside the hive (nectar, pollen, plants, honeydew secretions, air, water, soil, dust, etc.). Thus, the microbiota of bee products of diverse botanical and geographical origins differs. Secondary sources of the bee microbiome, such as humans and equipment involved in harvesting and post-harvest product manipulation, have also been identified^11,12^.

This study aimed to isolate and identify microorganisms from honey and bee bread harvested from Polish apiaries as efficient producers of enzymes with potential industrial use, namely glycoside hydrolases (amylases, cellulases, β-galactosidases and xylanases), proteases, and lipolytic enzymes (lipases and esterases). Furthermore, *Paenibacillus* sp. 8, an efficient producer of glycoside hydrolases, served as the source of *bgaP* gene, which was cloned and expressed in *Escherichia coli*. The recombinant BgaP β-galactosidase was purified and characterized.

## Results

### Isolation and identification of microorganisms from bee products and screening for various enzymatic activities

A total of 162 bacterial isolates, 21 yeast isolates, and two filamentous fungal isolates were obtained from ten honey samples of various botanical origins (nectar honey and honeydew honey). All honeys were sources of bacteria, while yeasts and fungi were isolated from acacia (13 yeast isolates), dandelion (7 yeast isolates), buckwheat (2 fungal isolates), nectar honeys, and coniferous honeydew honey (1 yeast isolate). Moreover, a bee bread sample was a source of 25 bacterial isolates, one yeast isolate, and one mold isolate. All isolates were cryopreserved as glycerol stocks at -80 °C and deposited in the local microbial culture collection at Gdansk University of Technology (GUT).

Microorganisms were grouped according to morphological features, namely macroscopic characteristics of the colonies and microscopic view of the cells, and enzymatic activities, such as amylolytic, cellulolytic, esterolytic, lipolytic, proteolytic, and β-galactosidase activity. Selected microorganisms were also tested for their ability to hydrolyze xylan.

Most yeast isolates did not show any enzymatic activity. Yeast strains 4, 5, 6, 11, and 13 hydrolyzed Tween 20 only, although the activity was quite weak. Fungal isolate no. 67, originating from buckwheat honey, formed brown mycelia and stained the culture fluid brown. It showed weak esterase activity towards glyceryl tributyrate (Tributyrin). In contrast, filamentous fungus no. P2 isolated from bee bread exhibited lipolytic activity on agar plates supplemented with Tween 20 and Tween 80, and it also efficiently hydrolyzed carboxymethylcellulose (CMC) and xylans from corncobs and beech wood. None of the eukaryotic microorganisms showed proteolytic, amylolytic, or β-galactosidase activities. Morphologically and biochemically different isolates were then subjected to taxonomic identification based on the sequence of the D1/D2 region of the large-subunit rDNA and the ITS1-5.8 S-ITS2 sequence, which revealed that yeasts belonging to *Starmerella magnoliae*, *Sporobolomyces johnsonii* and *Zygosaccharomyces siamensis*, and filamentous fungi were *Penicillium* sp. (*P. chrysogenum* or *P. tardochrysogenum*) and *Melnikomyces* sp. (*M. vietnamensis* or *M. thailandicus*) (see Supplementary Table S1 online). *S. magnoliae* strains were isolated from acacia and dandelion nectar honeys, as well as from bee bread, but only those from dandelion honey had lipolytic activity.

The most prevalent enzymatic activity exhibited by bacteria is proteolytic activity. A total of 95 bacterial strains from honey (59%) and 19 strains from bee bread (76%) hydrolyzed casein. Moreover, a significant number of bacteria produced esterases and lipases: 51 isolates from honey (31%) and 9 from bee bread (36%) hydrolyzed Tributyrin, 47 isolates from honey (29%) and 5 from bee bread (20%) hydrolyzed Tween 20, and 36 isolates from honey (22%) and 4 from bee bread (16%) hydrolyzed Tween 80. Additionally, cellulolytic activity was very often observed among bacteria originating from bee bread, that is, 64% of the isolates (16 out of 25) hydrolyzed CMC. In contrast, only 18% of the bacterial isolates from honey showed this activity (30 out of 162). The activities of other glycoside hydrolases were observed less frequently. Starch was hydrolyzed by 12 isolates from honey (7%) and 2 from bee bread (8%), and β-galactosidase activity was demonstrated by 2 bacterial isolates from bee bread (8%) and 5 from honey (3%). Bacteria obtained from the acacia honey sample did not show any of the tested enzymatic activities. Additionally, bacteria isolated from bee bread were tested for xylanolytic activity, and only one isolate no. P19 was capable of degrading xylans from corncobs and beech wood. Some morphologically and biochemically different isolates were subjected to 16 S rRNA gene sequencing (see Supplementary Table S2, online). The enzymatic activities of the isolates are presented in Supplementary Table S3.

Bacteria of the *Micrococcus* genus were most frequently isolated from almost all samples, but their properties differed. All isolates formed small, circular, convex, and opaque colonies on Luria-Bertani (LB-agar) plates after three days of incubation at 25 °C, but they were yellow (isolates no. 33, 55, 77, 176, 186, and P5), creamy yellow (isolate no. 44), creamy white (isolate no. 63), or white (isolates no. 83, 149, 187, 208, and P26). All isolates showed proteolytic activity, and some strongly hydrolyzed casein. Many isolates belonging to the *Micrococcus* genus exhibited esterolytic and lipolytic activities, whereas only *Micrococcus* sp. 186 weakly hydrolyzed starch (see Supplementary Table S3 online). The lipolytic bacteria, *Moraxella osloensis* no. 51 and *Acinetobacter lwoffii* no. 59, were isolated from honeydew honey. Moreover, the P11 isolate from bee bread, identified as *Peribacillus simplex*, showed only lipolytic activity (see Supplementary Table S3 online). Amylolytic activity was demonstrated by all isolates of the *Paenibacillus* genus, as well as *Metabacillus idriensis* no. 22, *Bacillus subtilis* no. P3, and *Bacillus* sp. 164 (see Supplementary Table S3, online). Additionally, bacteria belonging to the *Paenibacillus* genus showed significant cellulolytic activity. CMC was hydrolyzed by *B. subtilis* no. P3, *Bacillus* sp. strains 7, 23, 172, and P23; and isolate no. P7 is closely related to *Oceanobacillus profundus* (see Supplementary Table S3 online). Moreover, bacteria of the genera *Paenibacillus*, *Niallia circulans* (formerly *Bacillus circulans*), and isolate no. P8, closely related to *Cytobacillus dafuensis* hydrolyzes the chromogenic lactose analog 5-bromo-4-chloro-3-indolyl-β-d-galactopyranoside (X-gal). Because *Paenibacillus* sp. 8 exhibited the highest β-galactosidase activity, it was selected for further research.

### General characterization of the *Paenibacillus* sp. 8

Bacterial strain 8 was isolated from a dandelion honey sample. On LB agar plates, it produced round, flat, semi-transparent, and glossy yellowish colonies up to 2 mm in diameter. Cells of bacterium no. 8 were Gram-negative, rod-shaped, 0.9–1.2 μm wide and 3.5–5.5 μm long. Aerobic growth of strain 8 was observed at 25 °C and 30 °C, with optimum at 30 °C. It grew poorly at 37 °C, and growth was completely inhibited at 40 °C. The bacterium was able to grow at pH 5.8 and 7.9, with optimum at pH 6.9. Moreover, good growth was observed in YPSuc medium containing up to 10% sucrose. Partial growth inhibition occurred in the medium with 20% sucrose and complete inhibition occurred at 40% sugar concentration.

A comparison of the 16 S rRNA gene partial sequence (1475 bp) of bacterium no. 8 with that available in the GenBank database at the National Center for Biotechnology Information (NCBI) indicated that it belongs to the *Paenibacillus* genus. Strain no. 8 is most closely related to *P. amylolyticus* strain SQR-21 and *P. xylanexedens* strain PAMC 22,703 (see Supplementary Table S2 online), namely, 1474/1475 bp. Pairwise Sequence Alignment of the 16 S rRNA gene sequences of *Paenibacillus* sp. 8 and *Paenibacillus* sp. P19 (see Supplementary Table S2 online) showed 99.39% identity (1466/1475 bp) (EMBOSS Needle 1 tool^13^). Therefore, *Paenibacillus* sp. 8 was tested for xylanolytic activity on agar plates supplemented with xylans from beech wood and corncobs. After staining the plates with Congo Red solution, transparent halos appeared around the colonies, which indicated positive results (see Supplementary Table S3 online).

### Production of β-galactosidase in the *Paenibacillus* sp. 8

**Fig. 1:**
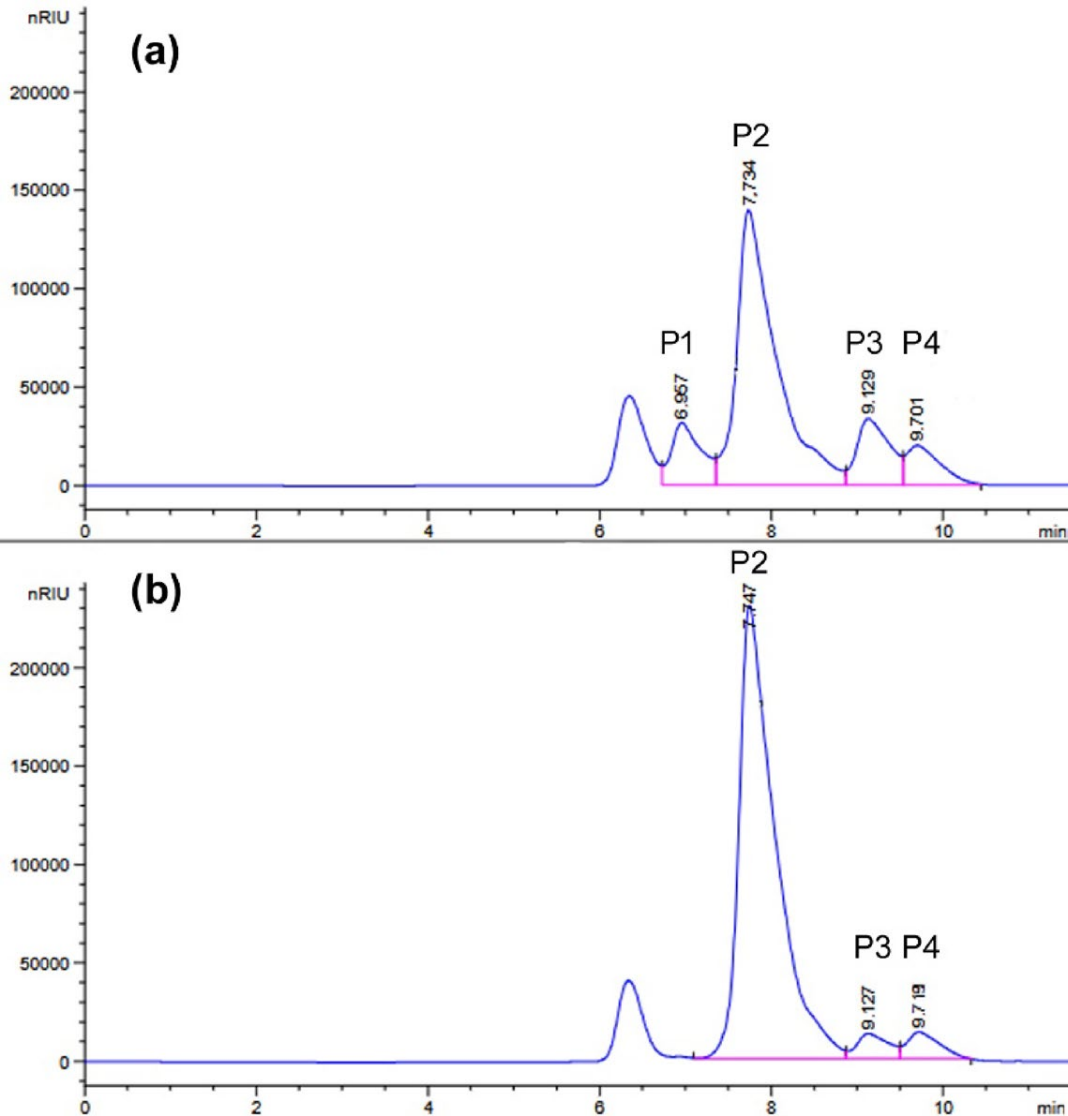
HPLC analysis using an Aminex HPX-87 H column of the products of milk lactose hydrolysis at 10 °C for 24 h, catalyzed by the cell-free extract of *Paenibacillus* sp. 8 with 0.97 U_ONPG_ of β-galactosidase activity per 1 mL of milk (**a**) and by the recombinant BgaP β-galactosidase at 125.7 U_ONPG_ per 1 mL of milk (**b**). The P1 peak in the chromatogram with a retention time of 6.957 min corresponds to GOS. Peaks P2, P3 and P4 correspond to lactose (t_R_ = 7.736 min), glucose (t_R_ = 9.137 min) and galactose (t_R_ = 9.731 min), respectively.

In the first stage, β-galactosidase production was performed in *Paenibacillus* sp. 8 cells. Bacteria were cultured in Luria-Bertani medium at 30 °C, and the expression of β-galactosidase-encoding genes was induced by addition of isopropyl β-d-1-thiogalactopyranoside (IPTG) or lactose. The *Paenibacillus* sp. 8 culture without induction served as a control. The β-galactosidase production was conducted for 24 h. Then, the enzyme activity in cell-free extracts was determined with synthetic substrate *o*-nitrophenyl-β-d-galactopyranoside (ONPG) at 30 °C and pH 6.6, and it was 4.8, 1.2 and 0.1 U/mL of IPTG-induced, control (without induction) and lactose-induced culture, respectively. The β-galactosidase activity in cell-free extracts was also confirmed using lactose as a substrate under the same conditions and it was 0.28, 0.09 and 0.01 U/mL of IPTG-induced, non-induced and lactose-induced cell-free extract, respectively. Moreover, lactose in milk was hydrolyzed by the addition of IPTG-induced cell-free extract of *Paenibacillus* sp. 8 to cow milk (volume ratio 2:8) and incubated at 10 °C for 24 h. The reaction products were determined using HPLC. As shown in Fig. 1a, lactose was partially hydrolyzed (efficiency of 27.3%), the released glucose remained in the solution, and over one-third of the released galactose (37.9%) was used for the synthesis of galactooligosaccharides (GOS).

### Identification of the β-galactosidase gene by constructing the genomic library of *Paenibacillus* sp. 8

The *Paenibacillus* sp. 8 genomic library was prepared using the pBAD/*Myc*-His A plasmid and *E. coli* strain TOP10 with a defective *lacZ* gene. After transformation, bacterial cells were plated on a selection medium containing ampicillin, an inducer of β-galactosidase gene expression, and a chromogenic β-galactosidase substrate X-gal, releasing a blue product upon hydrolysis. Among approximately 3,500 *E. coli* colonies, only one was blue. The recombinant 8Lib1 plasmid isolated from the blue colony contained a *Paenibacillus* sp. 8 genomic DNA fragment of approximately 8 kb. Since bacterial genes encoding β-galactosidase do not exceed 3.5 kbp, the 8Lib1 plasmid was subjected to size reduction of the genomic insert. The obtained 8Lib1.1 plasmid was reduced by approximately 1.5 kbp without damaging the β-galactosidase gene. Sanger sequencing and analysis of the 8Lib1.1 genomic insert revealed the sequence of *Paenibacillus* sp. 8 β-galactosidase gene. The *bgaP* gene sequence was deposited in the NCBI GenBank database and assigned accession no. PQ130282. An open reading frame of 2028 bp showed 98% identity with β-galactosidase genes from *P. xylanexedens* strain PAMC 22,703 (GenBank, genome sequence ID: CP018620.1, range: 3,283,275–3,285,302) and *P. amylolyticus* strain SQR-21 (GenBank, chromosome sequence ID: CP107037.1, range: 3,099,560–3,101,587). This ORF encoded a 675 amino acid protein with a calculated molecular mass of 77,020.03 Da and a theoretical pI of 5.12 (ProtParam tool on the ExPASy Proteomics Server^14^). A homology search performed using the BLASTP 2.16.1 program at the NCBI^15^ showed 670/675 (99.26%) amino acid identities and 673/675 (99.70%) positive for *P. xylanexedens* strain PAMC 22,703 β-galactosidase and 668/675 (98.96%) identities and 673/675 (99.70%) positive for the *P. amylolyticus* strain SQR-21 enzyme. Importantly, *P. xylanexedens* and *P. amylolyticus* β-galactosidase coding sequences were obtained only by automated computational analysis using the gene prediction method. The genomic insert of the 8Lib1.1 plasmid also contained a 431 bp fragment of the gene encoding the AraC family transcriptional regulator (GenBank accession no. WFA87837.1) on the complementary strand. Analysis of the 8Lib1.1 insert sequence with ProPr: Prokaryote Promoter Prediction v2.0 tool (http://ppp.molgenrug.nl/) revealed the presence of two promoters in the intergenic region on the + and - strands. Hence, the expression of *Paenibacillus* sp. 8 *bgaP* gene in *E. coli* TOP10 cells transformed with 8Lib1 and 8Lib1.1 plasmids occurred from its own promoter.

An analysis of the BgaP amino acid sequence performed using the InterPro 98.0 database at the EMBL’s European Bioinformatics Institute^16^ revealed that the *Paenibacillus* sp. 8 β-galactosidase is a member of the Glycoside Hydrolase family 42 (GH42). It is a three-domain protein consisting of a Glycoside hydrolase family 42 N-terminal domain (Glyco_hydro_42_N, residues 14–384), a β-galactosidase trimerization domain (Glyco_hydro_42_M, residues 398–607), and a β-galactosidase C-terminal domain (Glyco_hydro_42_C, residues 616–674).

A homology search, performed using the FASTA 1 program at the EMBL-EBI^13^, showed the highest 68.7% amino acid identity and 82.8% similarity with β-galactosidase from the alkaliphilic mesophyll *Niallia circulans* subsp. *alkalophilus* (formerly *Bacillus circulans* sp. *alkalophilus*) (RCSB PDB, accession no. 3TTS)^17^. *Paenibacillus* sp. 8 BgaP also showed high homology with β-galactosidases from psychrotolerant and halotolerant bacteria, *Planococcus* sp. L4 (GenBank accession no. ABI64125)^18^, and *Planococcus* sp. SOS Orange (GenBank accession no. AAF75984)^19^, with a 68.5 and 67.7% amino acid identity, respectively.

### Production and purification of the recombinant BgaP β-galactosidase

Expression of the *bgaP* gene was performed in *E. coli* TOP10 cells transformed with the 8Lib1.1 plasmid at 30 °C for 24 h. IPTG was added to one culture, while the other was left without the addition of the *bgaP* expression inducer. The BgaP β-galactosidase activity against ONPG determined in cell-free extracts at 40 °C and pH 6.6 was 5.9 ± 0.4 and 2.8 ± 0.2 U/mL for cultures with and without IPTG, respectively. The addition of IPTG was not necessary to achieve *bgaP* expression, although the expression level was twofold higher in the presence of the inducer.

**Fig. 2:**
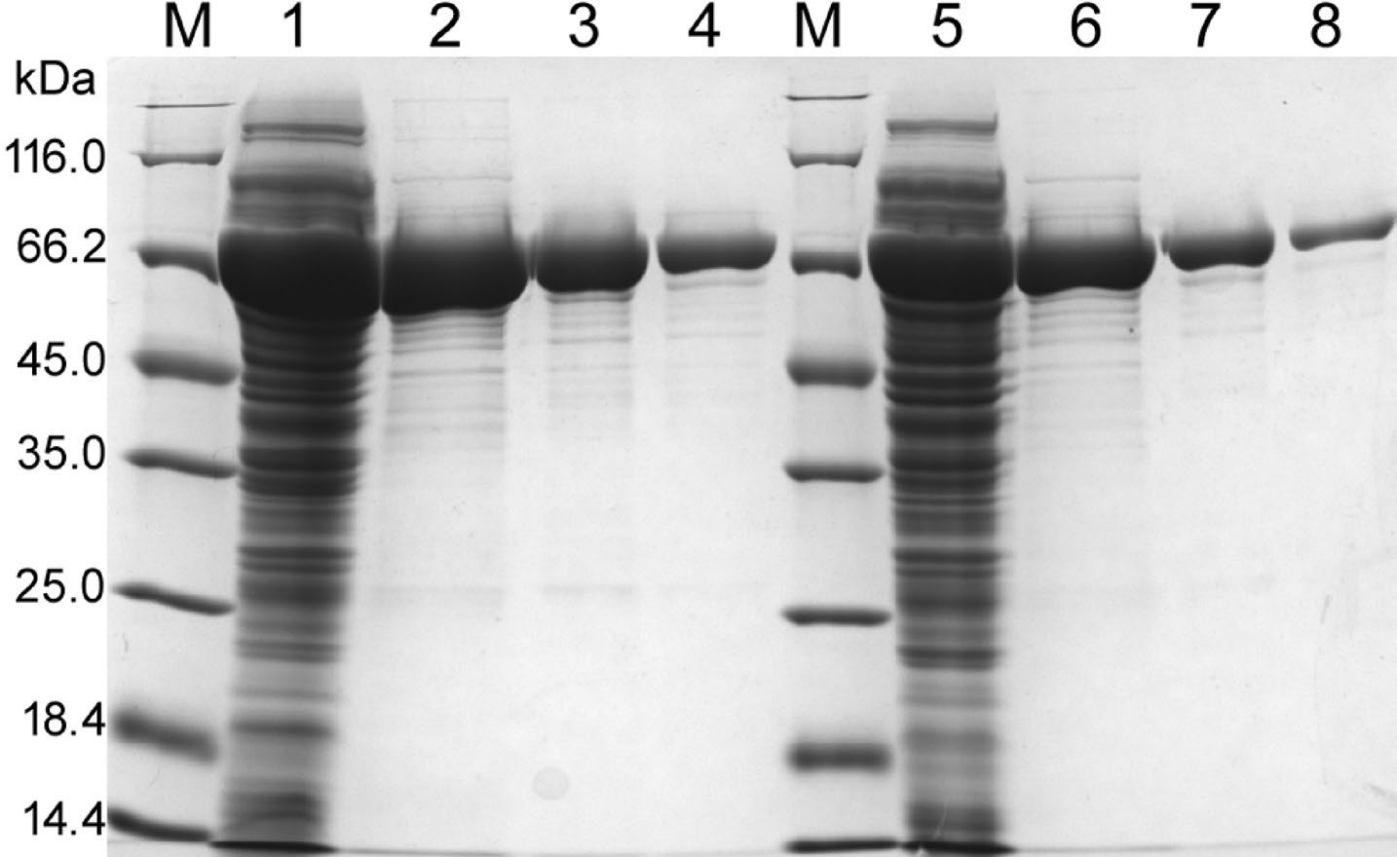
SDS-PAGE analysis of the fractions obtained by the *bgaP* gene expression in *E. coli* TOP10 cells and purification of the BgaP β-galactosidase. Lane M - Unstained Protein Molecular Weight Marker (Thermo Fisher Scientific Baltics, Vilnius, Lithuania), lanes 1 and 5 – cell-free extracts of *E. coli* TOP10 + pBAD/*bgaP* after *bgaP* gene expression at 30 and 37 °C, respectively; lanes 2 and 6 – purified BgaP protein after ion exchange chromatography; lanes 3 and 7 – purified BgaP protein after gel filtration (elution fractions no. 25); lanes 4 and 8 – BgaP protein after gel filtration (elution fractions no. 26).

To obtain more efficient production of the recombinant *Paenibacillus* sp. 8 β-galactosidase, the *bgaP* gene was PCR-amplified and cloned into the pBAD/*Myc*-His B vector under the control of the l-arabinose-inducible *ara*BAD promoter (P_BAD_). Heterologous gene expression was conducted in *E. coli* TOP10 cells at optimal temperatures for *E. coli* and *Paenibacillus* sp. 8 growth, namely, 37 °C and 30 °C. Recombinant BgaP β-galactosidase was purified using ion exchange chromatography (IEX) and gel filtration (GF). Sodium dodecyl sulfate-polyacrylamide gel electrophoresis (SDS-PAGE) confirmed the presence of a protein with a molecular weight of approximately 70 kDa in cell-free extracts and elution fractions from IEX and GF. Furthermore, it was shown that the recombinant β-galactosidase was produced more efficiently at 30 °C (Fig. 2). The BgaP activity against ONPG determined in cell-free extracts at 40 °C and pH 6.6 was 1257 ± 186 and 538 ± 62 U/mL for *E. coli* TOP10 transformed with pBAD/*bgaP* cultivated at 30 °C and 37 °C, respectively. As a result of heterologous production and purification, an enzyme with a specific activity of 1089 ± 14 U/mg was obtained. The molecular weight of the native enzyme, as estimated by GF, was 466,460.64 Da, suggesting that the *Paenibacillus* sp. strain 8 β-galactosidase has a hexameric structure in the solution.

### Structure prediction of the *Paenibacillus* sp. 8 BgaP β-galactosidase

**Fig. 3:**
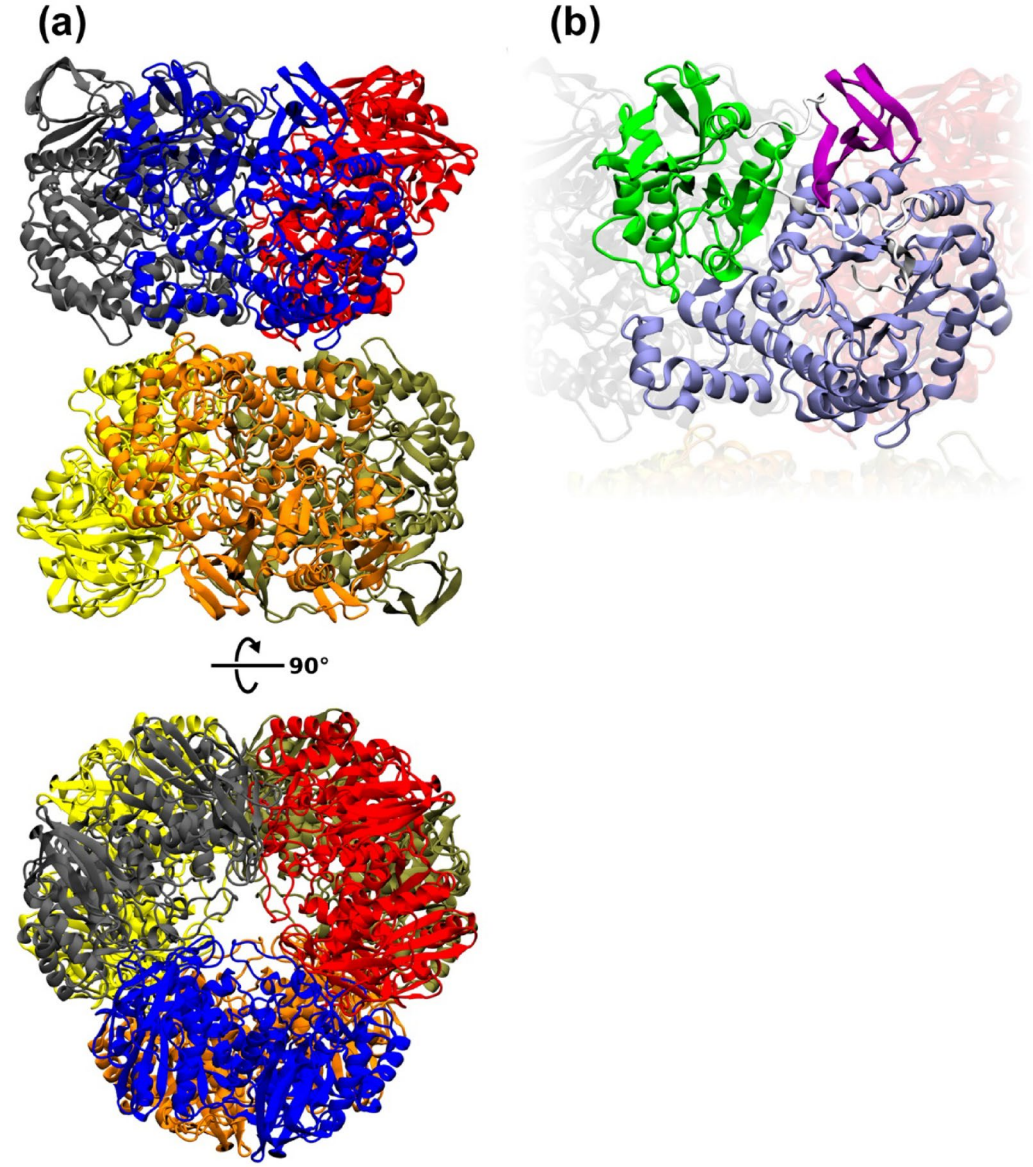
The 3D prediction of the *Paenibacillus* sp. 8 BgaP protein structure obtained with AlphaFold2. (**a**) Hexameric structure of the BgaP β-galactosidase. Each monomer was colored with a different color: grey, blue, red, yellow, orange and olive green. (**b**) The predicted monomer of the BgaP was colored according to the conserved domains. The Glycoside Hydrolase family 42 N-terminal domain (residues 14–384) was colored violet-blue, the β-galactosidase trimerization domain (residues 398–607) was colored green, and the β-galactosidase C-terminal domain (residues 616–674) was colored purple.

Protein structure prediction, carried out using the AlphaFold2 open-source code^20^, was used to determine the three-dimensional shape of the *Paenibacillus* sp. 8 BgaP enzyme from its amino acid sequence (GenBank accession no. XED97098) (Fig. 3a, b). The obtained structure prediction was compared with 3D structures from the RCSB Protein Data Bank, and the template modeling (TM) score was calculated using the tmtools 0.2.0 python package^21^. *Paenibacillus* sp. 8 BgaP was compared with the β-galactosidase from *N. circulans* subsp. *alkalophilus* (RCSB PDB, accession no. 3TTS)^17^, and the TM score was 0.995.

### Properties of the recombinant BgaP β-galactosidase

**Fig. 4:**
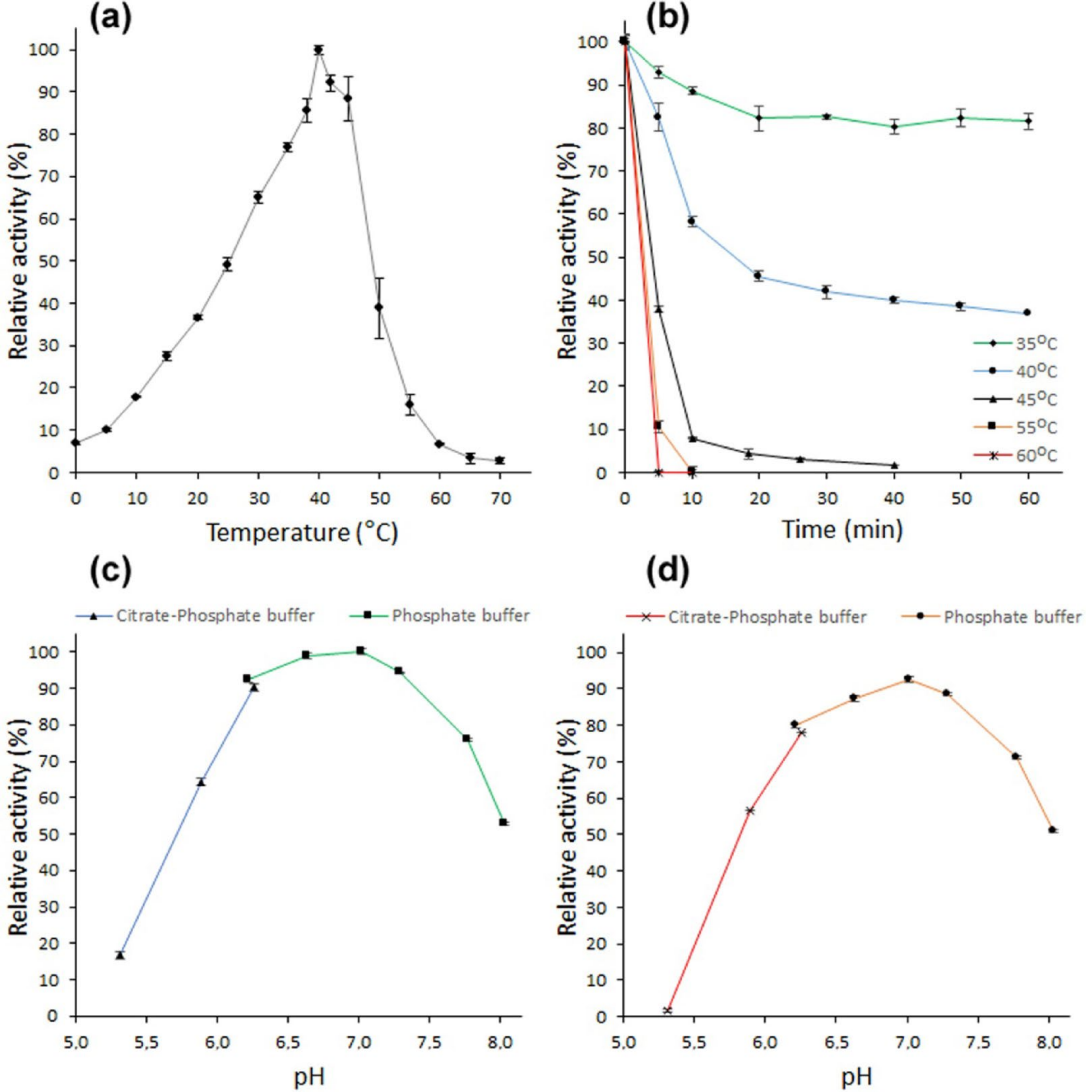
Biochemical characterization of the recombinant BgaP β-galactosidase. The effect of temperature on activity (**a**) and stability (**b**) of the enzyme was determined in 50 mM sodium phosphate buffer (pH 6.6), with ONPG as a substrate. For stability studies, the BgaP was incubated at 35–60 °C for 5–60 min and then assayed at 40 °C. The effect of pH on BgaP activity (**c**) and stability (**d**) was studied by measuring the β-galactosidase activity in 50 mM citrate-sodium phosphate buffers (pH 5.3–6.3) and 50 mM sodium phosphate buffers (pH 6.2-8.0) at 40 °C. For stability studies, the enzyme was diluted in buffers of appropriate pH, incubated at 10 °C for 24 h, and then assayed with ONPG at 40 °C.

The highest hydrolytic activity of recombinant *Paenibacillus* sp. 8 β-galactosidase with ONPG as a substrate was observed at 40 °C. The enzyme exhibited 49% maximum activity at 25 °C and maintained 7–18% of the maximum activity in a temperature range of 0–10 °C. At 70 °C, the β-galactosidase activity was only 3% of its maximum value (Fig. 4a). After 60 min of incubation at 35 and 40 °C, BgaP lost 19 and 63% of its initial activity, respectively. At 45 °C, 92% of the initial activity was lost within an incubation period of 10 min, and the BgaP enzyme was inactivated within 10 min at 55 °C or 5 min at 60 °C (Fig. 4b). The time required for BgaP β-galactosidase to lose half of its initial activity at 40 °C was 15.6 min, and at 45 °C the activity half-life (*t*_1/2_) decreased to 2.8 min.

To determine the optimum pH for the hydrolytic activity of the recombinant BgaP β-galactosidase, the enzyme was assayed with ONPG in a pH range of 5.3-8.0, at 40 °C. It exhibited over 50% of the maximum activity in a pH range of 5.9-8.0 with optimum at pH 6.6-7.0 (99–100% activity) (Fig. 4c). Furthermore, after 24 h of incubation at pH 6.6 and 7.0, BgaP lost 12 and 7% of its initial activity, respectively (Fig. 4d).

To examine the metal ion requirements, purified BgaP β-galactosidase was assayed in the presence of ethylenediaminetetraacetic acid sodium salt (EDTA), which is a chelator of divalent cations, as well as in the presence of selected monovalent, divalent, and trivalent metal ions. The enzyme was activated by K^+^, Na^+^, Li^+^, Mg^2+^ and Ca^2+^ and inhibited by Mn^2+^, Co^2+^, Ni^2+^, Cu^2+^ and Fe^3+^ ions, whereas EDTA had no effect on enzyme activity. Moreover, reducing compounds such as cysteine and reduced glutathione partially inhibited *Paenibacillus* sp. 8 β-galactosidase, whereas tris(2-carboxyethyl)phosphine hydrochloride (TCEP) completely inactivated it (Table 1).

**Table 1:** Effects of metal ions and selected reagents on the recombinant *Paenibacillus* sp. 8 β-galactosidase activity.

Metal ions (5 mM)	Relative activity (%)	Metal ions (10 mM)	Relative activity (%)	Reagents (10 mM)	Relative activity (%)
None	100	None	100	None	100
K^+^	105.2 ± 1.3	K^+^	110.1 ± 0.4	EDTA	99.7 ± 2.8
Na^+^	104.2 ± 0.2	Na^+^	108.4 ± 1.2	DTT	97.4 ± 2.3
Li^+^	102.7 ± 3.9	Li^+^	109.3 ± 1.5	Cysteine	59.8 ± 5.1
Mg^2+^	106.9 ± 6.2	Mg^2+^	112.6 ± 0.6	Glutathione (red.)	52.0 ± 1.3
Ca^2+^	111.4 ± 1.4	Ca^2+^	111.5 ± 1.2	TCEP	0.3 ± 0.3
Mn^2+^	33.6 ± 4.3	Mn^2+^	30.0 ± 4.6		
Co^2+^	36.6 ± 0.7	Co^2+^	23.0 ± 1.0		
Ni^2+^	16.5 ± 0.4	Ni^2+^	10.0 ± 0.1		
Cu^2+^	4.9 ± 0.9	Cu^2+^	3.3 ± 0.6		
Fe^3+^	6.8 ± 0.5	Fe^3+^	0.7 ± 0.1		

The BgaP activity was measured with 2.7 mM ONPG as a substrate.

Substrate specificity studies showed that BgaP glycoside hydrolase is active only against β-d-galactosides. Of the 10 chromogenic substrates tested, only *p*-nitrophenyl-β-d-galactopyranoside was hydrolyzed (see Supplementary Table S4 online).

The kinetic parameters of ONPG and lactose hydrolysis catalyzed by *Paenibacillus* sp. 8 β-galactosidase were determined in the temperature range of 10–40 °C and the values increased as the temperature increased. The *K*_m_ values with ONPG were 3.00, 3.62 and 4.51 mM at 10, 25 and 40 °C, respectively, while the *K*_m_ values with lactose were 51.76 and 55.50 mM at 10 and 30 °C, respectively. The *V*_max_ values for ONPG were 444.44, 1279.11 and 2444.04 U/mg at 10, 25, and 40 °C, respectively, while the *V*_max_ values for lactose were 0.204 and 1.252 U/mg at 10 and 30 °C, respectively. The *k*_cat_ values with ONPG were 570.41, 1641.65 and 3136.76 s^-1^ at 10, 25 and 40 °C, respectively, while the *k*_cat_ values with lactose were 0.262 and 1.607 s^-1^ at 10 and 30 °C, respectively. The *k*_cat_/*K*_m_ values for ONPG were 190.14, 453.40 and 695.02 s^-1^ mM^-1^ at 10, 25, and 40 °C, respectively, while the *k*_cat_/*K*_m_ values for lactose were 0.005 and 0.029 s^-1^ mM^-1^ at 10 and 30 °C, respectively. In general, the *K*_m_ values for ONPG were several times lower than those for lactose, indicating a much higher affinity of the enzyme for the synthetic substrate, and the BgaP β-galactosidase exhibited very high catalytic efficiency (*k*_cat_/*K*_m_) towards ONPG and very low affinity towards the natural disaccharide lactose.

BgaP activity towards ONPG was inhibited by the products of lactose hydrolysis, glucose, and galactose, with a much stronger inhibition by galactose (Table 2). In the case of fructose, a product of lactulose hydrolysis, the inhibition was weak, even at a high concentration of monosaccharides (Table 2). The *K*_i_ values determined at 40 °C using ONPG as a substrate were 27.33 and 6.99 mM for glucose and galactose, respectively. Both monosaccharides were competitive inhibitors, given that *V*_max_ values were unchanged, while *K*_m_ values increased from 4.51 to 5.63 and 7.30 mM for glucose and galactose, respectively. The *K*_i_ value for galactose, determined at 30 °C using lactose as a substrate was 6.96 mM, and the *K*_m_ value increased from 55.50 to 76.53 mM.

**Table 2:** Effects of galactose, glucose and fructose on the recombinant *Paenibacillus* sp. 8 β-galactosidase activity.

Sugar	Concentration (mM)	Relative activity (%)
None	0	100
Fructose	20	95.2 ± 0.4
	50	92.8 ± 1.2
	100	85.8 ± 0.8
Glucose	20	81.1 ± 4.7
	50	77.1 ± 2.2
	100	63.6 ± 4.7
Galactose	20	26.8 ± 7.0
	50	11.3 ± 3.6
	100	7.6 ± 2.3
Fructose + galactose	10 + 10	53.2 ± 0.1
	25 + 25	32.1 ± 0.1
	50 + 50	18.9 ± 0.2
Glucose + galactose	10 + 10	51.1 ± 4.7
	25 + 25	24.6 ± 1.8
	50 + 50	14.1 ± 2.2

The BgaP activity was measured with 2.7 mM ONPG as a substrate.

The hydrolysis of lactose in milk (120 mM) by the recombinant BgaP enzyme (125.7 U_ONPG_/mL) at 10 °C for 24 h revealed that only 8.6% of disaccharide was hydrolyzed and no GOS were formed (Fig. 1b).

**Fig. 5:**
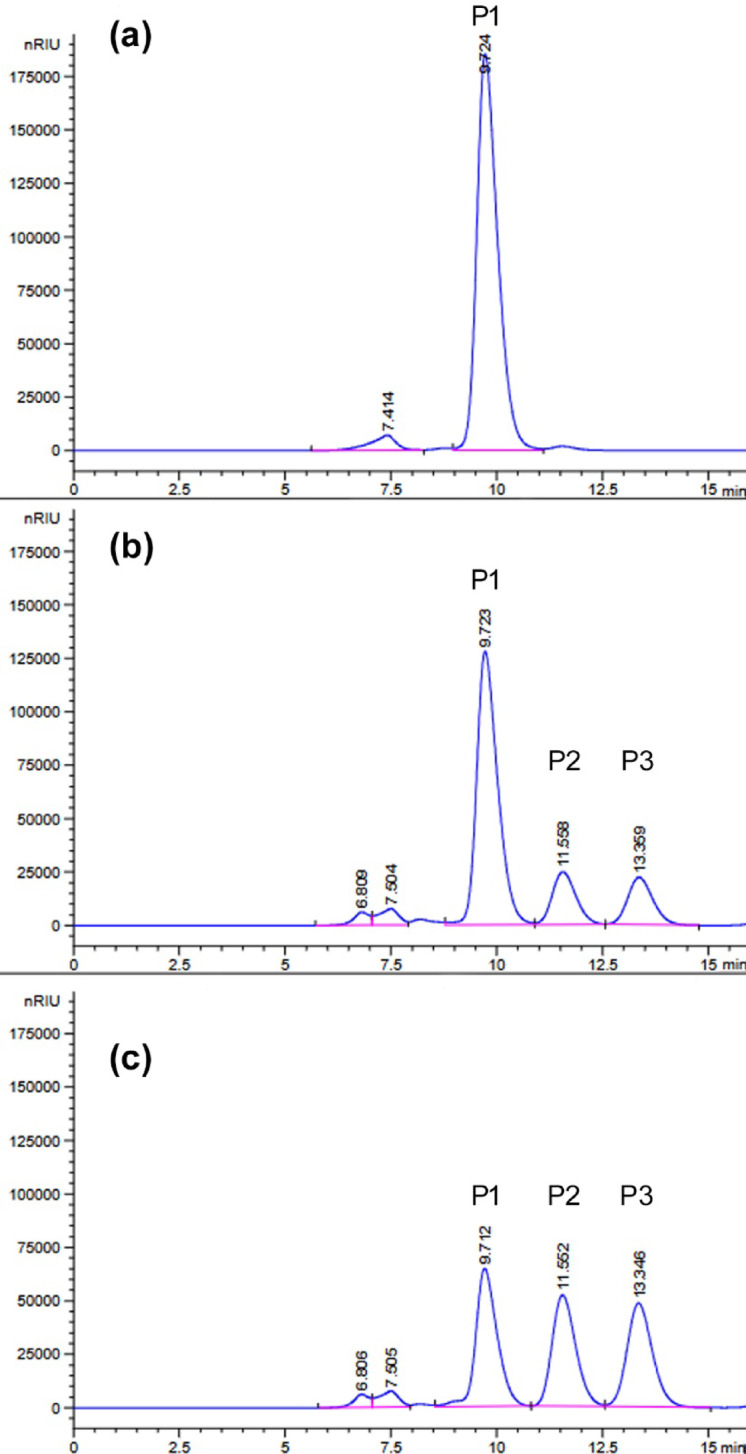
HPLC analysis using an Aminex HPX-87 C column of the products of lactulose hydrolysis catalyzed by the BgaP β-galactosidase (15.1 U_ONPG_/mL). (**a**) 120 mM buffered lactulose solution incubated at 30 °C for 24 h as negative control. (**b**) The reaction mixture after incubation at 10 °C for 24 h. (**c**) The reaction mixture after incubation at 30 °C for 24 h. Peaks P1, P2 and P3 in the chromatograms correspond to lactulose (t_R_ = 9.725 min), galactose (t_R_ = 11.551 min) and fructose (t_R_ = 13.336 min), respectively.

Another disaccharide, lactulose, was digested significantly better. After 24 h of the recombinant β-galactosidase (15.1 U_ONPG_/mL) incubation in 120 mM buffered lactulose solution at 10 °C, the hydrolysis efficiency was 31.1%, and at 30 °C it reached 63.8% (Fig. 5b, c). In the absence of BgaP in the reaction mixture, no lactulose hydrolysis products were detected after 24 h of incubation at 30 °C (Fig. 5a).

**Fig. 6:**
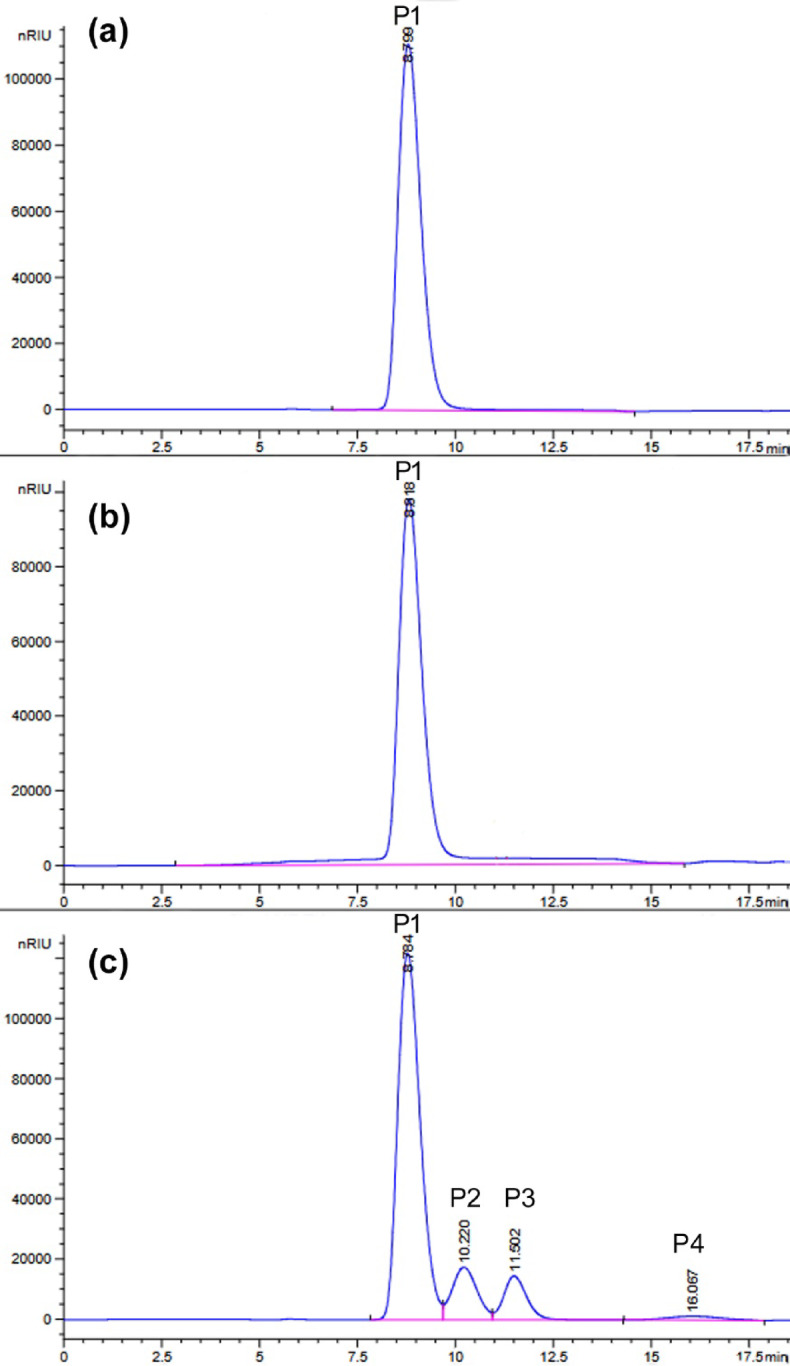
HPLC analysis using an Aminex HPX-87 C column of the products of lactose hydrolysis at 10 °C for 24 h, catalyzed by recombinant BgaP enzyme in the presence of recombinant l-arabinose isomerase from *Arthrobacter* sp. 22c. (**a**) The reaction mixture contained the BgaP β-galactosidase only (15.1 U_ONPG_/ mL). (**b**) The reaction mixture contained the AraA enzyme only (0.22 mg/mL). (**c**) The reaction mixture contained both enzymes. Peaks P1, P2, P3 and P4 in the chromatograms correspond to lactose (t_R_ = 8.801 min), glucose (t_R_ = 10.269 min), galactose (t_R_ = 11.551 min) and tagatose (t_R_ = 16.103 min), respectively.

**Fig. 7:**
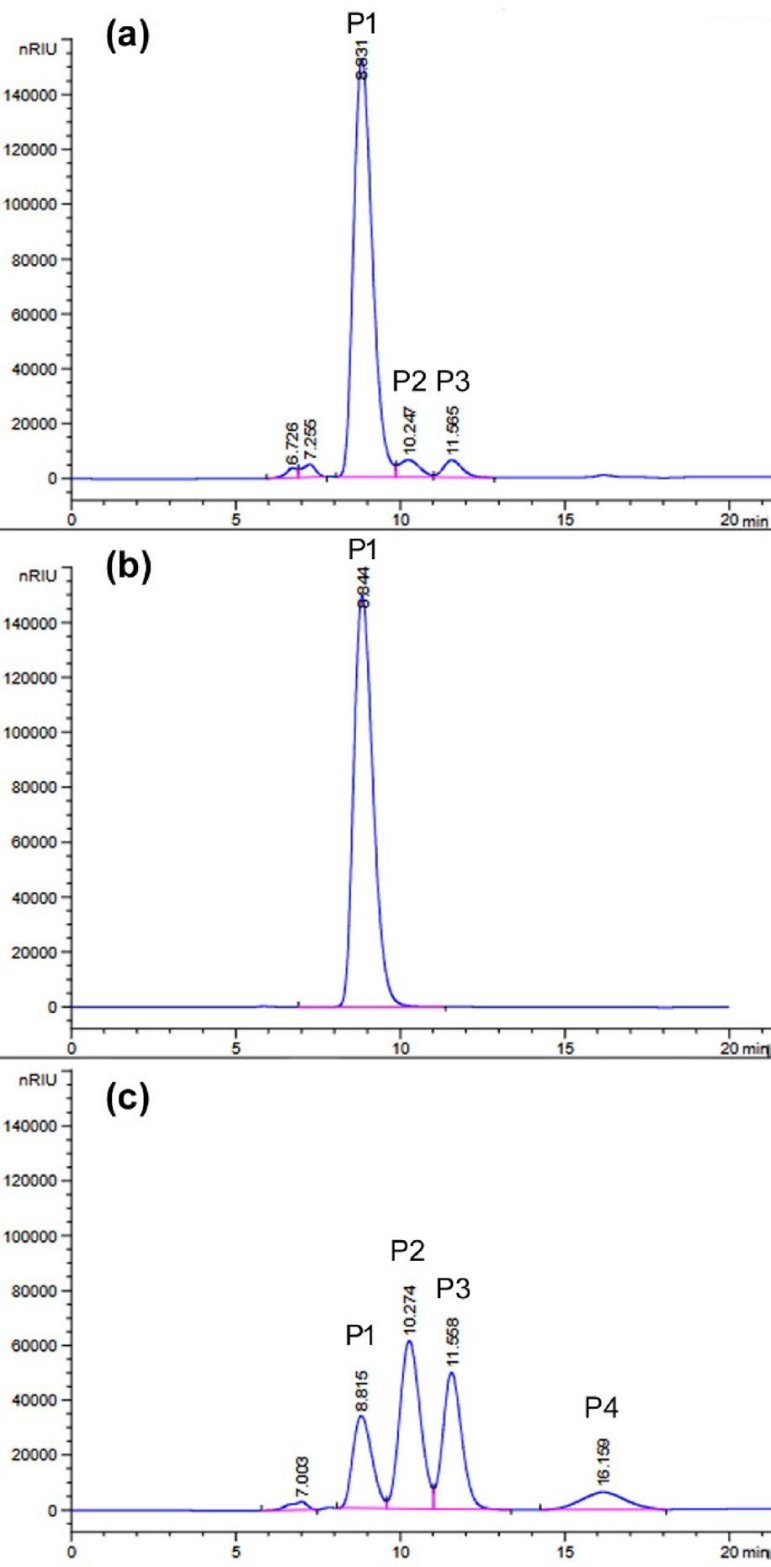
HPLC analysis using an Aminex HPX-87 C column of the products of lactose hydrolysis at 30 °C for 24 h, catalyzed by recombinant BgaP enzyme in the presence of recombinant l-arabinose isomerase from *Arthrobacter* sp. 22c. (**a**) The reaction mixture contained the BgaP β-galactosidase only (15.1 U_ONPG_/ mL). (**b**) The reaction mixture contained the AraA enzyme only (0.22 mg/mL). (**c**) The reaction mixture contained both enzymes. Peaks P1, P2, P3 and P4 in the chromatograms correspond to lactose (t_R_ = 8.801 min), glucose (t_R_ = 10.269 min), galactose (t_R_ = 11.551 min) and tagatose (t_R_ = 16.103 min), respectively.

The addition of recombinant l-arabinose isomerase from the *Arthrobacter* sp. strain 22c (0.22 mg/mL)^22^ to the reaction mixture containing 120 mM lactose and the BgaP β-galactosidase (15.1 U_ONPG_/mL) increased the lactose hydrolysis efficiency to 22.5 and 78.6% after 24 h of incubation at 10 and 30 °C, respectively (Figs. 6c and 7c). In the absence of AraA enzyme, which catalyzes the bioconversion of galactose to tagatose, only 7.4% of lactose was hydrolyzed within 24 h at 30 °C (Fig. 7a), and at 10 °C, no digestion products were detected (Fig. 6a). The absence of lactose hydrolysis products was also noted in the reaction mixtures containing l-arabinose isomerase alone (Figs. 6b and 7b), which were used as controls.

## Discussion

In our study, culture-dependent methods were employed to study the microbiota of honey and bee bread, and their enzymatic activities. The cultivation conditions, namely, a quite low temperature of 25 °C, a long incubation period of up to 5 days, and two types of culture media (LB agar and YPD agar), allowed us to obtain a wide range of aerobic bacteria, yeast, and filamentous fungi. Other researchers have grown bacteria at 37 °C for 24 h^23–25^ or at 30 °C for 5 days^26,27^.

Bee bread showed the highest microbial diversity. Of the ten honey samples tested, dandelion honey (a unifloral nectar honey collected in spring, creamy yellow in color, and delicate flavor) had the highest biodiversity. In general, bee products such as honey and bee bread are unfavorable environments for the development of microorganisms, so they contain mainly spores instead of vegetative forms. Therefore, bacteria belonging to the Bacillaceae family, *Bacillus* spp., and related genera, as well as *Paenibacillus* spp. that produce endospores, are the most frequently isolated^23–25,27^. The isolation of non-endospore-forming bacteria belonging to the *Micrococcus* and *Acinetobacter* has been less frequently reported. Pomastowski et al.^25^ isolated *M. luteus* H24 from Polish rape nectar honey and *Micrococcus* sp. H32 (closely related to *M. aloeverae* AE-6, *M. yunnanensis* YIM65004, and *M. luteus* NCTC2665) from Australian leatherwood honey. *M. yunnanensis* CTB32 and *A. lwoffii* CT110 were isolated from Greek honey samples^27^. Moreover, *M. luteus* D2 has been isolated from propolis^26^. According to Donkersley et al.^28^ and Laconi et al.^29^, *Acinetobacter* spp. are abundant in honey, pollen, and bee bread samples investigated using culture-independent methods. *Acinetobacter* spp. and *Micrococcus* spp. in bee products come from the environment, namely *Acinetobacter* from soil, and *Micrococcus* from the air, dust, plants, pollen, and honeydew secretions. Moreover, bacteria of the *Micrococcus* genus are typical colonizers of the hive and have also been recorded in larval and adult bee gastrointestinal tracts^11^. In our study, we also obtained one bacterial isolate, *Moraxella osloensis*. According to the literature, *Moraxella* spp. have previously been isolated from the digestive tracts of honey bees collected from hives in eastern Slovakia^30^.

In addition to bacteria, beehive products contain yeast. Pollen stored by honey bees undergoes fermentation to bee bread carried out by lactic acid bacteria and yeasts; thus, various yeast species have been isolated from bee bread samples, and most of the isolates belong to the *Starmerella* and *Zygosaccharomyces* genera^31–33^. *S. apis* was the most frequent and abundant species in fresh bee bread, followed by *S. magnoliae* (formerly *Candida magnoliae*, *Torulopsis magnoliae*), *S. apicola* (formerly *C. apicola*), and *S. bombicola*. Aged bee bread is dominated by *Zygosaccharomyces* spp., including *Z. siamensis*. *Starmerella* spp. and *Zygosaccharomyces* spp. were also detected in dissected honey stomachs. Moreover, *S. magnoliae*, *Z. mellis*, *Z. rouxii*, and *Z. siamensis* were isolated from unripened honey^32^. Ziuzia et al. isolated *S. magnoliae* and *Yarrowia lipolytica* from Polish lime honey^34^. Research conducted by Gilliam showed that *S. magnoliae* (*T. magnoliae*) was present in bee-collected pollen but not in flower pollen^35^. Together, these results indicated that *S. magnoliae* originated from the bee microbiome. Additionally, from a honeydew honey sample, we isolated a yeast strain closely related to *Sporobolomyces johnsonii*, which is consistent with reports that yeasts of the *Sporobolomyces* genus originate from environments such as plants, pollen, or honeydew^11^.

Other microorganisms commonly found in bee products, such as honey, pollen, and bee bread, are filamentous fungi, and the genera *Alternaria*, *Aspergillus*, *Cladosporium* and *Penicillium* have been most frequently isolated^36–38^, while the *Melnikomyces* genus has not yet been reported.

The abundance and diversity of beehive product microbiota confirmed by many studies have allowed scientists to assume that they could be good sources of microorganisms with great application potential. Therefore, the antimicrobial activity of bacteria isolated from honey, bee-collected pollen, and bee bread has been studied in recent years. The best producers of antimicrobial compounds are bacteria belonging to the genus *Bacillus* and related genera such as *Paenibacillus* and *Lactobacillus*^23,24,27,39,40^. In contrast, *Bacillus* spp. and related genera originating from various environments are known to produce and secrete many macromolecule-degrading enzymes such as glycoside hydrolases, proteases, and lipolytic enzymes^41–44^. However, little is known about the enzymatic activity of the beehive product microbiota. Research conducted by Pełka et al. showed that of 81 bacterial strains isolated from pollen and bee bread samples, 69 had lipolytic, 54 proteolytic, 39 cellulolytic, and 29 esterolytic activities. Moreover, 11 isolates exhibited weak β-galactosidase activity, and only 3 had amylolytic activity. Most isolates showed several enzymatic activities that are characteristic of bacteria belonging to the *Bacillus* genus^24^. α-Amylase-producing *B. atrophaeus* NRC1 was isolated from commercial Egyptian honey. AmyI, an attractive enzyme for the baking industry, has been produced, purified, and characterized^45^. Moreover, three *Acinetobacter* strains isolated from hive-stored bee bread in China showed proteolytic and lipolytic activities^36^. In our study, *Paenibacillus* sp. 8 was selected as an efficient producer of glycoside hydrolases, whereas the better producers of proteases and lipases were isolates belonging to the *Micrococcus* genus and Moraxellaceae family (*Moraxella osloensis* and *Acinetobacter lwoffii*), respectively. The proteolytic activity of *M. aloeverae* and related species has been previously described^46,47^.

Bacteria belonging to the genus *Paenibacillus* are widely distributed in nature, from the polar regions to the tropics. Most of these species have been isolated from soil, especially the rhizosphere, but *Paenibacillus* spp. have also been found in fresh and sea water, sediments, compost, and sewage. The genus *Paenibacillus* includes more than 200 species. Some species are pathogens of invertebrates, for example, *P. larvae* cause honeybee disease American foulbrood (AFB). *Paenibacillus* spp. has also been isolated from human clinical samples^48,49^. Bacteria of the genus *Paenibacillus* are aerobic or facultatively anaerobic, rod-shaped, Gram-positive, Gram-negative, Gram-variable, non-pigmented, and endospore-forming bacteria. Endospores can survive extreme conditions, such as high temperature, pressure and UV irradiation, allowing them to withstand pasteurization. Moreover, many strains of *Paenibacillus* can grow at refrigerated temperatures, causing spoilage of both raw and pasteurized milk^48^. In our study, *Paenibacillus* strains were isolated from honey and bee bread samples, in which they most likely occurred as spores because *Paenibacillus* sp. 8 was unable to grow at low pH and high sugar concentrations. The pH values of honey are usually between 3.5 and 5.5, and the concentration of carbohydrates ranges from 70 to 80% (w/w)^50^. However, honey is often added as a sweetener to food, where bacteria can grow and spoil the food.

In contrast, *Paenibacillus* spp. produce many enzymes of industrial importance^48^. *Paenibacillus* sp. 8, such as *P. amylolyticus* strain NRRL NRS-290^T^ and *P. xylanexedens* strain B22a^T^, has enzymes capable of degrading plant cell-wall polysaccharides such as cellulose and xylan, can hydrolyze lactose (β-galactosidase activity), Tween 20, but not Tween 80^51,52^. Moreover, it efficiently hydrolyzes starch and has weak proteolytic activity towards casein, similar to *P. amylolyticus* strain NRRL NRS-290^T^^51^.

Whole cells of *Paenibacillus* sp. 8 or a mixture of secreted glycoside hydrolases (cellulase, xylanase, and amylase) can potentially be used to degrade plant biomass to produce substrates (oligo-and monosaccharides) for various applications in the food and beverage, feed, and biofuel industries. Some of the applications of *Paenibacillus* spp. and their enzymes have already been patented^48^. However, little is known about β-galactosidases from bacteria of the *Paenibacillus* genus and their suitability for lactose hydrolysis and oligosaccharide synthesis. Therefore, in this study, we investigated the β-galactosidase from *Paenibacillus* sp. 8.

Multiple sequence alignment of *Paenibacillus* sp. 8 BgaP with its counterparts from mesophilic *N. circulans* subsp. *alkalophilus*^17^ and psychrotolerant *Planococcus* sp. L4^18^, performed using the EMBL-EBI Clustal Omega 1 program^53^, revealed some conserved regions (see Supplementary Fig. S1 online), four cysteine residues of the metal-binding site (C115, C155, C157, and C160)^17^, and two essential catalytic residues, namely E150 and E307, corresponding to E150 and E307 of *N. circulans* subsp. *alkalophilus* β-galactosidase and identified as an acid/base catalyst and a nucleophile^17^, and E151 and E308 of *Planococcus* sp. L4 enzyme (see Supplementary Fig. S1 online).

The asymmetric unit of a *N. circulans* subsp. *alkalophilus* β-galactosidase crystal contains six monomers, although the authors suggested that the enzyme can form stable trimers in solution and form a hexamer only at high protein concentrations^17^. The BgaP β-galactosidase from *Paenibacillus* sp. 8 characterized in this study has a hexameric structure in solution, even at low concentrations such as 0.1 mg/mL. The hexameric quaternary arrangement was also noted for GH42 β-galactosidase from the psychrophilic *Marinomonas* sp. ef1^54^, while β-galactosidases from *Planococcus* sp. strains L4 and SOS Orange were dimers in their native forms^18,19^. Other members of the GH family 42, such as cold-adapted β-galactosidases from *Arthrobacter* sp. 32c and *Rahnella* sp. R3 forms trimers in solution^55,56^, and six *Rahnella* sp. R3 β-galactosidase molecules constitute two trimers in a crystallographic asymmetric unit^57^.

The BgaP β-galactosidase from *Paenibacillus* sp. 8 contains all the amino acid residues involved in the binding of α-galactose, identified in the galactose-soaked crystals of *N. circulans* subsp. *alkalophilus*, that is, R111, N149, E150, E307, Q313, E355 and H358, which form hydrogen bonds with α-galactose, and W187, which forms hydrogen bonds with α-galactose via a water molecule^17^ (see Supplementary Fig. S1 online). Binding of galactose in the α-anomeric form can explain the strong competitive inhibition of the BgaP enzyme by galactose. When hydrolysis is completed and the β-anomer of galactose is released, it undergoes mutarotation in solution and can bind to the active site of the enzyme as an α-anomer.

The temperature-activity profile and low thermal stability indicate that the BgaP β-galactosidase from *Paenibacillus* sp. 8 is more similar to enzymes originating from psychrotolerant bacteria, such as β-galactosidase from *Planococcus* sp. SOS Orange, which showed maximum activity at 42 °C, approximately 20% of maximum activity at 10 °C, and was inactivated within 10 min of incubation at 55 °C^19^, or β-galactosidase from *Arthrobacter* sp. 32c with maximum activity at 50 °C, and approximately 20% of maximum activity at 10 °C^55^, compared to the mesophilic counterpart *N. circulans* subsp. *alkalophilus*, which was maximally active at 55 °C and retained 13% of its maximum activity at 10 °C^17^. The hexameric GH42 β-galactosidase from an Antarctic bacterium *Marinomonas* sp. ef1 was maximally active at 55 °C, retained 23% of its maximum activity at 5 °C, and was stable for several days at 37 °C. According to the authors, the hexameric quaternary structure and the absence of a Zn^2+^ binding site may allow the β-galactosidase from *Marinomonas* sp. ef1 to function at low temperatures^54^. The BgaP β-galactosidase characterized in this study has the features of a cold-adapted enzyme and a hexameric structure; however, it also has a metal ion-binding site conserved in β-galactosidases belonging to the GH family 42. The Zn^2+^ binding cluster comprising four Cys residues has been found in β-galactosidases from the thermophilic bacteria *T. thermophilus* A4^58^ and *G. stearothermophilus* T6^59^, the mesophilic bacterium *N. circulans* subsp. *alkalophilus*^17^, and the psychrophilic bacterium *Rahnella* sp. R3^57^. Zinc ions were present in all the aforementioned crystal structures, with one Zn^2+^ ion per protein monomer. According to the authors, Zn^2+^ does not participate in the enzymatic reaction but stabilizes the catalytic domain. This is consistent with the results of biochemical studies, which showed that GH42 β-galactosidases such as Gan42B from *G. stearothermophilus* T6 or R-β-Gal from *Rahnella* sp. R3 does not require metal ions for its activity^57,59^. Moreover, the chelating agent EDTA did not affect the catalytic activity of the Gan42B protein, β-galactosidases from *Planococcus* sp. strain L4 and SOS Orange, and *Arthrobacter* sp. 32c enzyme^18,19,55,59^, as well as the BgaP enzyme from *Paenibacillus* sp. 8 characterized in this study.

The addition of Mg^2+^ and Ca^2+^ ions slightly activated β-galactosidases from *Rahnella* sp. R3^56^ and *Paenibacillus* sp. 8, but had no effect on enzymes from *Planococcus* sp. strain L4 and SOS Orange^18,19^. The activation of β-galactosidases with calcium ions is particularly important because it can potentially increase the efficiency of lactose hydrolysis in milk, whey, and whey permeate. On the other hand, Ni^2+^ ions significantly reduce the activity of β-galactosidases^18,19,55^, including enzyme from *Paenibacillus* sp. 8; therefore, we used IEX and GF to purify the BgaP protein instead of the frequently used Ni-affinity chromatography.

The optimal pH for BgaP activity was close to the values reported for other GH42 β-galactosidases because the enzymes from *Planococcus* sp. SOS Orange, *Arthrobacter* sp. 32c, and *Rahnella* sp. R3 are maximally active against ONPG at pH 6.5^19,55,56^, β-galactosidase from *Planococcus* sp. L4 at pH 6.8^18^, and the enzyme from *N. circulans* subsp. *alkalophilus* in a wide pH range, from 6.0 to 7.5^17^. The maximum activity of β-galactosidase from *Marinomonas* sp. ef1 was noted at pH 6.0, but the enzyme showed 80% of the maximum activity in the pH range of 5.0–7.0^54^.

β-Galactosidases belonging to the GH family 42 are highly active against chromogenic β-d-galactosides, although some enzymes have slightly broader substrate specificities. β-Galactosidase from *Planococcus* sp. L4 was active only against ONPG and PNPG^18^, similar to the BgaP enzyme from *Paenibacillus* sp. 8, whereas the β-galactosidase from *Planococcus* sp. SOS Orange showed weak activity against *o*NP- and *p*NP-β-d-fucopyranosides^19^, and the enzyme from *Arthrobacter* sp. 32c digested *o*NP- and *p*NP-β-d-glucopyranosides^55^.

GH42 β-galactosidases have a greater affinity for ONPG than for lactose, and *K*_m_ values for a synthetic substrate usually do not exceed 10 mM. For example, *K*_m_ values determined at 10 °C were 3.8, 4.5 and 5.75 mM for enzymes from *Planococcus* sp. L4, *Planococcus* sp. SOS Orange and *Arthrobacter* sp. 32c, respectively^18,19,55^. At the same temperature, the *K*_m_ values determined for lactose were: 11.2 and 77.54 mM for *Planococcus* sp. L4 and *Arthrobacter* sp. 32c β-galactosidases, respectively^18,55^. This resulted in higher catalytic efficiencies (*k*_cat_/*K*_m_) of 51.3, 23.0 and 9.12 s^− 1^ mM^− 1^ for ONPG^18,19,55^ compared to 5.5 and 0.023 s^− 1^ mM^− 1^ for lactose^18,55^. The exception was the β-galactosidase from *Rahnella* sp. R3, which has a greater affinity for lactose than ONPG with *K*_m_ values of 2.2 and 6.5 mM, respectively; and similar *k*_cat_/*K*_m_ values of 1.1 and 1.2 s^− 1^ mM^− 1^ (at 4 °C)^56^. Unfortunately, the catalytic efficiency of the BgaP enzyme from *Paenibacillus* sp. 8 towards lactose was 4.6 times lower than that reported for *Arthrobacter* sp. 32c β-galactosidase^55^, although the *k*_cat_/*K*_m_ value for ONPG was much higher than the values reported for all the above-mentioned β-galactosidases belonging to the GH family 42.

The weak affinity for lactose and inhibition of BgaP β-galactosidase by both galactose and glucose resulted in poor hydrolysis of the disaccharide in milk. Stronger inhibition with galactose than with glucose was also observed for the enzyme from *T. thermophilus* A4 (formerly *Thermus* sp. A4). In the presence of 50 mM galactose, the relative activity of the enzyme towards ONPG was approximately 20%, whereas in the presence of 50 mM glucose, it was approximately 65%^60^. Cold-active β-galactosidase from *Planococcus* sp. L4 was slightly inhibited by galactose, with *K*_i_ value of 38 mM; thus, the enzyme efficiently hydrolyzed lactose in milk under refrigeration^18^. In contrast, the cold-adapted β-galactosidase from *Arthrobacter* sp. 32c was strongly inhibited by glucose, but the efficiency of lactose hydrolysis was increased by the addition of glucose isomerase to the reaction mixture, which converted glucose into fructose^55^. Similarly, the efficiency of lactose digestion by BgaP β-galactosidase from *Paenibacillus* sp. 8 was increased by the addition of l-arabinose isomerase from *Arthrobacter* sp. 22c^22^, which converted galactose into tagatose. In the presence of AraA, a tenfold increase in lactose hydrolysis efficiency at 30 °C was observed. Since tagatose is sweeter and less caloric than galactose and also has prebiotic properties^22^, this approach allows for obtaining a sweetener with health-promoting properties. In the future, we plan to test whey permeate as an inexpensive and abundant lactose-rich substrate for this purpose because the development of a production process for such a functional sweetener would allow the conversion of a dairy by-product into a value-added product. It is worth mentioning that the minerals contained in whey and whey permeate (Ca, Mg, Na, and K)^61^ have a positive effect on the activity of both enzymes, namely BgaP β-galactosidase from *Paenibacillus* sp. 8 and l-arabinose isomerase from *Arthrobacter* sp. 22c^22^.

It has been shown that β-galactosidases from the GH family 42 exhibit different activities towards natural β-galactosides. Some of them hydrolyze lactose efficiently, while others poorly or not at all. The β-galactosidase from *Exiguobacterium acetylicum* MF03 was able to hydrolyze lactulose (galactosyl-fructose) but was unable to hydrolyze lactose (galactosyl-glucose)^62^, and the Gan42B enzyme from *G. stearothermophilus* T-6 exhibited significant hydrolytic activity towards β-1,4-galactobiose (galactosyl-galactose) and short β-1,4 linked GOS, with no detectable activity against lactose^63^. The BgaP β-galactosidase from *Paenibacillus* sp. 8 digested lactulose nine times more efficiently than lactose at 30 °C; therefore, this enzyme can be useful for the formulation of lactulose detection and quantification kits, in which the first step is selective hydrolysis of this disaccharide. Furthermore, this result confirms that ketohexoses (fructose and tagatose) are weaker inhibitors of BgaP than aldohexoses (glucose and galactose).

GH42 β-galactosidases have different abilities to synthesize oligosaccharides. The enzyme from *N. circulans* sp. *alkalophilus* can both hydrolyze lactose and synthesize GOS in a reverse hydrolysis reaction^17^, and the β-galactosidase from *E. acetylicum* MF03 synthesizes fructosyl-galactooligosaccharides (fGOS) from lactulose^62^. In the case of BgaP enzyme from *Paenibacillus* sp. 8, however, no significant amounts of fGOS were detected in the reaction with lactulose, indicating that hydrolysis is the predominant activity of this β-galactosidase.

In contrast, the significant activity of the cell-free extract of *Paenibacillus* sp. 8 towards milk lactose, both hydrolysis and GOS synthesis, suggested the presence of a β-galactosidase other than BgaP in bacterial cells. A β-galactosidase belonging to GH family 2, with a molecular mass of 453.7 kDa and tetrameric quaternary structure, suitable for lactose hydrolysis and GOS synthesis was produced by a thermophilic *Paenibacillus barengoltzii* CAU904 isolated from the South China Sea^64^.

## Conclusions

The present results confirm that the composition of beehive products microbiota depends on their botanical and geographical origin; thus, our study expands the knowledge about the microbial communities associated with Polish honey and bee bread. Moreover, this study proves that beehive products can be valuable sources of whole-cell biocatalysts, raw enzyme mixtures, and individual catalytic proteins from various groups, such as proteases, lipases, and glycoside hydrolases, with potential industrial applications. The results also show that enzymes produced by microorganisms isolated from honey and bee bread can be the subject of both basic and applied research. The BgaP β-galactosidase obtained and characterized in this study, in combination with l-arabinose isomerase, has the potential to be applied to the utilization of lactose-rich dairy by-products for the production of functional sweeteners.

## Materials and methods

### Chemical reagents and ingredients of culture media

Peptone K, yeast extract and bacteriological agar were purchased from BTL (Łódź, Poland). Ampicillin sodium salt, chloramphenicol, carboxymethylcellulose sodium salt (CMC), l-(+)-arabinose, d-(-)-fructose, d-(+)-glucose, d-(+)-galactose, lactose monohydrate, lactulose, soluble starch, sucrose, yeast nitrogen base without amino acids (YNB), isopropyl-β-d-thiogalactopyranoside (IPTG), 5-bromo-4-chloro-3-indolyl-β-d-galactopyranoside (X-gal), *o*-nitrophenyl-β-d-galactopyranoside (ONPG), glyceryl tributyrate (Tributyrine), Tween 20, Tween 80, l-glutathione reduced, tris(2-carboxyethyl)phosphine hydrochloride (TCEP), dl-dithiothreitol, l-cysteine, ethylenediaminetetraacetic acid disodium salt dehydrate (EDTA) and 2-(N-morpholino)ethanesulfonic acid (MES) were supplied by Sigma (St. Louis, MO, USA). Xylan from beech wood was purchased from Apollo Scientific (Bredbury, Stockport, United Kingdom). Xylan from corncombs was supplied by Pol-Aura (Zawroty, Morąg, Poland). Congo red (0.5% solution) was purchased from Chempur (Piekary Śląskie, Poland). d-Tagatose was purchased from Molekula (München, Germany). All the other chemicals were supplied by POCH (Gliwice, Poland).

### Honey and bee bread samples

Samples of buckwheat (3 samples), multifloral (2 samples), honeydew, honeydew/buckwheat, and forest (honeydew/multifloral) honeys were provided by beekeepers from Pomeranian Voivodeship in northern Poland. Bee bread, acacia (*Robinia pseudoacaccia*), and dandelion honey were purchased from apiaries in the Kuyavian-Pomeranian Voivodeship in mid-northern Poland.

### Isolation of microorganisms

Honey samples were diluted with sterile room-temperature water in a mass ratio of 1:1. Bee bread was soaked in sterile water at a 1:9 ratio and vortexed. Subsequently, a lawn streaking method was performed as follows: 150 µL of each sample was spotted on five LB agar plates (1% peptone K, 1% NaCl, 0.5% yeast extract, and 1.5% bacteriological agar) for isolation of bacteria and five YPD-agar plates (2% peptone K, 1% yeast extract, 2% glucose, and 2% bacteriological agar) supplemented with ampicillin (100 µg/mL) and chloramphenicol (34 µg/mL) for isolation of yeasts and filamentous fungi. The plates were incubated for 3–5 days at 25 °C. Based on the colony morphology, each morphotype was transferred onto a fresh plate. For the long-term storage of pure cultures, glycerol stocks were prepared by mixing the liquid cultures with 40% glycerol in a volume ratio 1:1 and freezing at -80 °C.

### Enzymatic activity assays

Appropriate selective plates were prepared to test the enzymatic activity of the isolated strains. The plates were inoculated with microorganisms and incubated at 25 °C for up to 5 days. The β-galactosidase activity of the bacteria was investigated on LB agar plates supplemented with IPTG (79 µg/mL) and X-gal (20 µg/mL). For yeasts and fungi YPL agar plates containing 2% peptone K, 1% yeast extract, 2% lactose, 2% bacteriological agar, and supplemented with X-gal (20 µg/mL) were prepared. Strains capable of producing β-galactosidase grew as blue colonies. The lipolytic activity of bacteria was tested on modified LB agar plates containing 1% peptone K, 0.5% NaCl, 2% bacteriological agar, 0.01% CaCl_2_, and 1% (v/v) Tween 20 or Tween 80. Modified YPD agar plates (2% peptone K, 2% agar, and 1% glucose) with 0.01% CaCl_2_ and 1% (v/v) Tween 20 or Tween 80 were used to investigate yeasts and fungi. A cloudy zone around the colony indicated a positive result. Esterolytic activity was tested on modified LB agar medium (0.2% peptone K, 0.1% yeast extract, 1% NaCl, and 1.5% bacteriological agar) supplemented with 1% (v/v) Tributyrine and modified YPD agar medium (0.4% peptone K, 0.2% yeast extract, 2% glucose, and 1.5% bacteriological agar) supplemented with 1% (v/v) Tributyrine. The plates consisted of two layers: the bottom layer of pure medium and the upper layer of medium supplemented with Tributyrine. Strains capable of esterase production exhibited a clear zone surrounding the colony. Proteolytic activity was tested on modified LB agar plates (0.5% yeast extract, 0.2% peptone K, 1% NaCl, and 1.5% bacteriological agar) containing 2% (v/v) skim milk and modified YPD agar plates (0.4% peptone K, 1% yeast extract, 1.5% agar, and 2% glucose) with 2% (v/v) skim milk. A positive result was defined as a clear zone around the colony. The amylolytic activity of the bacteria was tested on LB agar plates supplemented with 1.5% starch. Yeast and fungal isolates were tested on YPS agar plates containing 2% peptone K, 1% yeast extract, 1.5% bacteriological agar, and 1.5% starch. After incubation, the plates were flooded with Gram’s iodine solution, turning the plate blue unless a strain capable of starch hydrolysis was present, in which case the colony was surrounded by a clear yellowish zone. Cellulolytic activity was tested on LB agar plates supplemented with 1.5% CMC or YPC agar plates (2% peptone K, 1% yeast extract, 1.5% bacteriological agar, 1.5% CMC). After incubation, the plates were flooded with 5 mL 0.1% Congo red solution and stained for 15 min at room temperature. Decolorization was then performed using 10 mL of 1 M NaCl for 15 min. Cellulase-producing strains were characterized by colorless zones around them. The production of bacterial xylanases was tested on EAX plates containing 0.25% yeast extract, 1.5% bacteriological agar, and 0.7% xylan from beech wood or corn cobs. Yeast and filamentous fungi were tested on YNBX agar plates containing 0.67% YNB, 0.7% xylan, and 1.5% bacteriological agar. After incubation, the plates were stained with a 0.1% Congo red solution, followed by decolorization with 1 M NaCl. A colorless halo around the colony indicated a positive result. To estimate the level of enzymatic activity of microbial isolates, excluding β-galactosidase activity, the diameters of the halo zones and bacterial colonies were measured and the Halo Zone Index (HI) was calculated using the following formula^65^:

$$\:\text{H}\text{a}\text{l}\text{o}\:\text{Z}\text{o}\text{n}\text{e}\:\text{I}\text{n}\text{d}\text{e}\text{x}\:\left(\text{H}\text{I}\right)=\frac{\text{H}\text{a}\text{l}\text{o}\:\text{z}\text{o}\text{n}\text{e}\:\text{d}\text{i}\text{a}\text{m}\text{e}\text{t}\text{e}\text{r}\:\text{w}\text{i}\text{t}\text{h}\:\text{c}\text{o}\text{l}\text{o}\text{n}\text{y}\:\text{d}\text{i}\text{a}\text{m}\text{e}\text{t}\text{e}\text{r}\:\left(\text{c}\text{m}\right)\:}{\text{C}\text{o}\text{l}\text{o}\text{n}\text{y}\:\text{d}\text{i}\text{a}\text{m}\text{e}\text{t}\text{e}\text{r}\:\left(\text{c}\text{m}\right)}$$

### Identification of microorganisms

Microorganisms that grew on YPD agar medium supplemented with antibiotics (ampicillin and chloramphenicol) were confirmed as either yeast or fungi through microscopic observation. Gram staining of the bacterial strains was performed using the Gram Kolor kit (Stamar, Dąbrowa Górnicza, Poland). To perform rDNA sequence-based identification, genomic DNA was isolated, and PCR amplification of bacterial 16S rDNA and the D1/D2 domain of the large-subunit rDNA and ITS1-5.8S-ITS2 fragment of yeast and fungal rDNA was carried out using PCR Mix Plus HGC (A&A Biotechnology, Gdynia, Poland). The Genomic Mini Kit (A&A Biotechnology, Gdynia, Poland) was used for DNA extraction from the bacteria. The DNA ExtractMe DNA yeast kit (Blirt, Gdańsk, Poland) was used for yeast DNA isolation, and the Bead-Beat Micro AX Gravity kit (A&A Biotechnology, Gdynia, Poland) was used for DNA extraction from filamentous fungi. PCR amplification of the 16S rDNA fragment was performed using the fD1 primer 5’ ccgaattcgtcgacaacAGAGTTTGATCCTGGCTCAG 3’ and the rP2 primer 5’ cccgggatccaagcttACGGCTACCTTGTTACGACTT 3’^66^. Linker sequences containing restriction sites for cloning were designated as lowercase letters. The D1/D2 region of LSU rDNA was amplified using primers NL1 (F63) 5’ GCATATCAATAAGCGGAGGAAAAG 3’ and NL4 (LR3) 5’ GGTCCGTGTTTCAAGACGG 3’, and the primers employed for the ITS1-5.8-ITS2 fragment of rDNA amplification were ITS1 5’ TCCGTAGGTGAACCTGCGG 3’ and ITS4 5’ TCCTCCGCTTATTGATATGC 3’^67^. Amplicons were purified using the DNA Clean-Up kit (A&A Biotechnology, Gdynia, Poland) and sequenced by Macrogen Europe (Amsterdam, The Netherlands). The 16S rDNA amplicons of isolates no. 8 and P19 were first cloned into a pUC19 plasmid and then sequenced by Genomed (Warsaw, Poland) using M13fwd 5’ GTAAAACGACGGCCAGT 3’ and M13rev 5’ CAGGAAACAGCTATGAC 3’ primers. PCR was performed using Hybrid PCR Master Mix (2x) supplied by EURx (Gdańsk, Poland) and genomic DNA isolated from bacterial strains no. 8 and P19 as a template. The blunt-ended PCR products were purified using a Clean-Up Kit (A&A Biotechnology, Gdynia, Poland) and cloned into the pUC19 plasmid (New England Biolabs, Ipswich, MA, USA) digested with *Sma*I restriction enzyme (Thermo Fisher Scientific Baltics, Vilnius, Lithuania) for 2 h at 30 °C. Ligation was carried out in a 20 µL reaction mixture containing 1 µL of T4 DNA ligase (400 U) purchased from New England Biolabs (Ipswich, MA, USA) for 1 h at room temperature (approximately 22 °C), and then the reaction was stopped by heat inactivation of the enzyme at 65 °C for 10 min. Both reaction mixtures were used to transform chemically competent *E. coli* TOP10 cells (Invitrogen, Carlsbad, CA, USA) containing a defective β-galactosidase gene. After transformation, *E. coli* cells were streaked on LB agar plates supplemented with ampicillin (100 µg/mL), X-gal (20 µg/mL), and IPTG (79 µg/mL), incubated at 37 °C for 24 h, and then screened for the presence of white colonies without β-galactosidase activity. The recombinant plasmids were isolated using a Plasmid Mini kit (A&A Biotechnology, Gdynia, Poland), subjected to analysis using *Eco*RI and *Hin*dIII restriction enzymes (Thermo Fisher Scientific Baltics, Vilnius, Lithuania) to estimate the size of the insert (approximately 1500 bp), and sequenced.

### Cultivation of the *Paenibacillus* sp. 8 in various conditions

Growth of *Paenibacillus* sp. 8 was assessed at temperatures of 25, 30, 37, and 40 °C on LB agar plates for 48 h. Growth rates at 30 °C were measured between pH 3.1 and 8.8 using buffered LB media (1% peptone K, 0.5% yeast extract, 1% NaCl) at pH 3.1, 4.0, 4.9, 5.8, 6.9, 7.9, and 8.8. The optical density of each 24 h culture was measured at 600 nm. The bacterial strain was also cultivated in YP (1% peptone K, 0.5% yeast extract) and YPSuc media containing 1, 5, 10, 20 and 40% sucrose, as an osmotic pressure increasing agent, at 30 °C with shaking at 160 rpm for 24 h. Then, the OD_600_ of each culture was measured.

### Production of wild-type β-galactosidase in the *Paenibacillus* sp. 8

To produce the wild-type enzyme with β-galactosidase activity, *Paenibacillus* sp. 8 was grown in 15 mL LB medium (1% peptone K, 0.5% yeast extract, 1% NaCl, pH 7.0) at 30 °C for 24 h with shaking at 160 rpm. Then, 4 mL of the culture was added to three 100 mL portions of fresh LB medium and further cultivated under the same conditions for 4 h. The expression of β-galactosidase was initiated by the addition of IPTG or lactose at a final concentration of 1 mM or 0.5%, respectively. The enzyme was produced for another 24 h, then the cultures were centrifuged (10 min, 5,000 × *g*, 4 °C), and the cell pellets were resuspended in 10 mL of 50 mM sodium phosphate buffer (pH 6.6). The third culture was performed without the addition of an inducer. *Paenibacillus* sp. 8 cells were disintegrated by sonication (5 cycles of 1 min each with 1 min breaks at a vibration amplitude of 5 μm), and the cell lysates were centrifuged at 12,000 × *g* for 10 min to obtain cell-free extracts. β-Galactosidase activity was then measured with ONPG (0.8 mg/mL) and lactose (25 mM) as substrates at pH 6.6 and 30 °C. One unit of the *Paenibacillus* sp. 8 β-galactosidase activity was defined as the quantity of enzyme releasing of 1 µmol *o*-nitrophenol or glucose per 1 min at 30 °C and pH 6.6. The amount of glucose released during lactose hydrolysis was estimated using the Glucose (GO) Assay Kit (Sigma-Aldrich, St. Louis, MO, USA) according to the manufacturer’s instructions.

### Preparation of the *Paenibacillus* sp. 8 genomic library

The pBAD/*Myc*-His A plasmid (Invitrogen, Carlsbad, CA, USA) lacking the 5’-terminal part of the *lacZ* gene encoding the N-terminal fragment of *E. coli* β-galactosidase was used to construct a genomic DNA library of *Paenibacillus* sp. 8. Genomic DNA from the frozen at -80 °C bacterial cell pellet was isolated using a Genomic Mini kit (A&A Biotechnology, Gdynia, Poland). Both genomic and plasmid DNA were digested with *Bgl*II and *Hin*dIII restriction enzymes in R buffer (Thermo Fisher Scientific Baltics, Vilnius, Lithuania) in 25 µL reaction mixtures containing 1 µL (10 U) of each enzyme for 2 h at 37 °C. The digested vector and genomic DNA fragments were purified using the Clean-Up Concentrator kit (A&A Biotechnology, Gdynia, Poland) and ligated using T4 DNA Ligase (New England Biolabs, Ipswich, MA, USA). Ligation was carried out in a 20 µL reaction mixture containing 1 µL of ligase (400 U) for 1 h at room temperature (about 22 °C), and then the reaction was stopped by heat inactivation of the enzyme at 65 °C for 10 min. In addition, the digested pBAD/*Myc*-His A vector was autoligated and used as a control. Both reaction mixtures were used to transform chemically competent *E. coli* TOP10 cells (Invitrogen, Carlsbad, CA, USA) with a defective β-galactosidase gene. After transformation, *E. coli* cells were streaked on LB agar plates containing ampicillin (100 µg/mL), X-gal (20 µg/mL), and IPTG (79 µg/mL), incubated at 37 °C for 24 h, and then screened for the presence of blue colonies with β-galactosidase activity. The recombinant plasmid was isolated using a Plasmid Mini kit (A&A Biotechnology, Gdynia, Poland) and subjected to analysis using *Bgl*II, *Hin*dIII, *Sal*I, and *Xho*I restriction enzymes (Thermo Fisher Scientific Baltics, Vilnius, Lithuania) to estimate the size of the insert.

To reduce the size of the genomic insert in the library plasmid, it was digested with the *Xho*I restriction enzyme. The digestion products were separated and purified, and the longer product was autoligated. After transformation, *E. coli* TOP10 cells were plated on LB agar medium supplemented with ampicillin, IPTG, and X-gal, and incubated at 37 °C for 24 h to confirm β-galactosidase activity.

### Production of the BgaP β-galactosidase in *E. coli* TOP10 cells carrying the 8Lib1.1 plasmid

To produce recombinant BgaP β-galactosidase, *E. coli* TOP10 cells (Invitrogen, Carlsbad, CA, USA) transformed with the 8Lib1.1 plasmid were grown in LB medium supplemented with ampicillin (100 µg/mL) at 37 °C for 18 h with shaking at 160 rpm. Then, 2 mL of the culture was added to two 100 mL portions of fresh LB medium with antibiotics and further cultivated under the same conditions for 2 h. Expression of the *bgaP* gene was initiated by the addition of IPTG to one of the two cultures, to a final concentration of 1 mM. The BgaP β-galactosidase was produced for another 22 h at 30 °C, then the cultures were centrifuged (5,000 × *g*, 10 min, 4 °C), and the cell pellets were resuspended in 10 mL of 50 mM sodium phosphate buffer (pH 6.6). The *E. coli* cells were sonicated (5 cycles of 1 min each with 1 min breaks at a vibration amplitude of 5 nm), and the cell lysates were centrifuged at 12,000 × *g* for 10 min to obtain cell-free extracts. BgaP β-galactosidase activity was then measured with ONPG at pH 6.6 and 40 °C.

### Construction of *E. coli* expression system for the production of BgaP β-galactosidase

The *bgaP* gene encoding *Paenibacillus* sp. 8 β-galactosidase was amplified using F8pBADNco 5’ gagt*ccATGG*TTAGTAACAAACTGCCCAAAATGTTCTACG 3’, and R8pBADGAPXba 5’ ctag*tctaga***TTA**TTTCATTTCGAGCAGTTGCACTC 3’ primers containing *Nco*I and *Xba*I recognition sites, respectively (italics). The start and stop codons are given in bold. PCR was performed using Hybrid PCR Master Mix (2x) purchased from EURx (Gdańsk, Poland) and *Paenibacillus* sp. 8 genomic DNA as a template. The 2044 bp PCR product was purified using a Clean-Up Kit (A&A Biotechnology, Gdynia, Poland), digested with *Nco*I and *Xba*I restriction enzymes (Thermo Fisher Scientific Baltics, Vilnius, Lithuania), and cloned into a pBAD/*Myc*-His B vector (Invitrogen, Carlsbad, CA, USA) digested with the same restriction enzymes. The obtained pBAD/*bgaP* recombinant plasmid contained the *bgaP* gene under the control of the *ara*BAD promoter (P_BAD_). Analysis of the recombinant plasmid with the *Pag*I (*Bsp*HI) restriction endonuclease, followed by Sanger sequencing, confirmed the correctness of its construction.

### Production of the BgaP β-galactosidase in *E. coli* TOP10 cells carrying the pBAD/*bgaP* plasmid and purification of the recombinant enzyme

*E. coli* TOP10 cells carrying the pBAD/*bgaP* plasmid were grown for approximately 20 h at 37 °C in LB medium supplemented with ampicillin (100 µg/mL) with shaking (160 rpm). Then, 2 mL of culture was added to two 100 mL portions of LB medium containing antibiotic and cultivated at 37 °C for 2 h. 20% solution of l-arabinose was then added to the final concentration of 0.1% and cultivation was continued for 22 h at 37 °C or 30 °C at 160 rpm. The cultures were then centrifuged (5,000 × *g*, 10 min, 4 °C), cell pellets were resuspended in 10 mL of buffer A (20 mM sodium phosphate buffer pH 6.6 containing 50 mM NaCl), and the cells were sonicated (5 cycles of 1 min each, with a vibration amplitude of 5 μm). After centrifugation (12,000 × *g*, 10 min, 4 °C), the supernatant was applied to a MonoQ™ HR 5/5 column (Amersham Biosciences, Uppsala, Sweden) equilibrated with buffer A. The column was washed with five volumes of buffer A and the recombinant enzyme was eluted with a linear gradient of sodium chloride (50-1050 mM) in the same buffer (20 volumes of the column). Fractions showing β-galactosidase activity with ONPG were pooled and loaded onto a Superdex™ 200 Increase 10/300 GL column (GE Healthcare Bio-Sciences, Uppsala, Sweden). Elution was performed using the same buffer (20 mM sodium phosphate buffer pH 6.6 containing 50 mM NaCl). Fractions containing BgaP β-galactosidase were pooled, mixed with 40% glycerol in a 1:1 ratio, and stored at 8 °C. The concentration of purified protein was determined spectrophotometrically at a wavelength of 280 nm. The molecular weight of the native BgaP enzyme was estimated using the MWGF 1000 Kit for Protein Molecular Weights 29,000-700,000 Da, purchased from Sigma (St. Louis, MO, USA).

### Characterization of the recombinant BgaP β-galactosidase

The activity of the purified BgaP β-galactosidase under various conditions was tested using the chromogenic substrate ONPG. The reaction mixture was prepared in a ratio 8:2 of ONPG solution (1 mg/mL of 50 mM sodium phosphate buffer, pH 6.6), and the enzyme was suspended in the same buffer. ONPG hydrolysis was stopped after 2 min by adding 1 M sodium carbonate to a final concentration of 230 mM. The absorbance of the mixture was measured at 410 nm. One unit of *Paenibacillus* sp. 8 β-galactosidase activity was defined as the quantity of enzyme releasing of 1 µmol *o*-nitrophenol per 1 min at 40 °C and pH 6.6. All experiments were performed at least three times. Data are presented as the mean ± SD.

#### Temperature optimum and thermal stability determination

The effect of temperature on BgaP enzyme activity was examined at temperatures ranging from 0 to 70 °C. The ONPG solution was pre-incubated at a specific temperature before adding the enzyme, hydrolysis of the substrate was carried out at the same temperature, and the reaction was stopped by adding sodium carbonate. For thermal stability studies, β-galactosidase was pre-incubated at various temperatures for different periods of time (from 5 to 60 min), and the residual activity was then measured at 40 °C. The thermal inactivation rate constant (*k*_d_) of the enzyme at specific temperature was calculated using the following equation:

$$\:\text{ln}\left({A}_{t}/{A}_{0}\right)=-{k}_{d}t$$

where *A*_t_ was the residual β-galactosidase activity at time *t*, and *A*_0_ was the initial BgaP activity in the thermally untreated control. The *k*_d_ value was determined by linear regression of ln(*A*_t_/*A*_0_) versus incubation time, with *k*_d_ corresponding to the slope. The enzyme activity half-life (*t*_1/2_) was calculated using the following formula:

$$\:{t}_{1/2}=\text{ln}\left(2\right)/{k}_{d}$$

#### Study of the influence of pH on the BgaP activity and stability

To determine the optimal pH for β-galactosidase activity, nine 1 mg/mL ONPG solutions were prepared in buffers with different pH ranging from 5.3 to 8.0 (50 mM citrate-phosphate buffer for pH 5.3 to 6.3 and 50 mM phosphate buffer for pH 6.2 to 8.0). BgaP was diluted in the same buffers. The enzyme was mixed with substrate in a 2:8 ratio and incubated for 2 min at 40 °C. Then, 1 M Na_2_CO_3_ was added to a final concentration of 230 mM and the absorbance at 410 nm was measured. Furthermore, the effect of pH on the stability of BgaP was assessed by incubating the enzyme dilutions at 10 °C for 24 h and then estimating the residual activity at 40 °C.

#### Study of the influence of metal ions and selected chemical compounds on the BgaP activity

To determine the effects of various metal ions on BgaP β-galactosidase, enzyme activity was examined at 40 °C with ONPG dissolved in 20 mM MES buffer pH 6.6 (1 mg/mL) in the presence of 5 or 10 mM KCl, NaCl, CH_3_COOLi*2 H_2_O, MgCl_2_*6 H_2_O, CuSO_4_*5 H_2_O, CaCl*2 H_2_O, NiCl_2_*6 H_2_O, MnCl_2_*4 H_2_O, CoCl_2_*6 H_2_O, and FeCl_3_*6 H_2_O. The effects of 10 mM EDTA, TCEP, DTT, l-cysteine, and l-glutathione (reduced) on BgaP activity were examined at 40 °C using ONPG dissolved in 20 mM sodium phosphate buffer pH 6.6. The inhibitory effects of glucose, galactose, and fructose on recombinant β-galactosidase were also determined using ONPG at pH 6.6 and 40 °C. The reaction mixtures contained a single carbohydrate at a concentration of 20–100 mM or an equimolar mixture of glucose and galactose or fructose and galactose at a final concentration of 20–100 mM.

#### Substrate specificity determination

The substrate specificity of BgaP enzyme was estimated using the following chromogenic compounds purchased from Sigma (St. Louis, MO, USA): *p*-nitrophenyl-β-d-galactopyranoside, *p*-nitrophenyl-α-d-galactopyranoside, *p*-nitrophenyl-β-d-glucopyranoside, *p*-nitrophenyl-α-d-glucopyranoside, *p*-nitrophenyl-β-d-fucopyranoside, *p*-nitrophenyl-β-d-xylopyranoside, *p*-nitrophenyl-β-d-glucuronide, *p*-nitrophenyl-β-d-mannopyranoside, *p*-nitrophenyl-β-d-cellobioside, and *p*-nitrophenyl-β-l-arabinofuranoside. Each substrate was dissolved in 20 mM sodium phosphate buffer pH 7.0 at a concentration of 1 mg/mL. Hydrolysis was carried out at 40 °C for 2 min, and then halted by the addition of 1 M sodium carbonate to a final concentration of 230 mM.

#### Kinetic studies

The kinetic parameters of ONPG and lactose hydrolysis were determined in 50 mM sodium phosphate buffer pH 6.6 containing 1–5 mM ONPG and 25–100 mM lactose at temperatures ranging from 10 to 40 °C. The amount of glucose released during lactose hydrolysis was estimated using the Glucose (GO) Assay Kit (Sigma-Aldrich, St. Louis, MO, USA) according to the manufacturer’s instructions. *K*_m_ and *V*_max_ values were obtained using the Lineweaver-Burk equation. The *K*_i_ values were determined at 40 °C using 5 mM galactose or glucose.

### Hydrolysis of lactose in milk

The hydrolysis of lactose in milk by β-galactosidase from *Paenibacillus* sp. 8 was performed at 10 °C for 24 h, similarly to the industrial production of low-lactose milk. The cell-free extract of *Paenibacillus* sp. 8 was mixed with cow’s milk (4.8% lactose, 3% protein, 2% fat) in a ratio of 2 to 8, giving 0.97 U_ONPG_ of β-galactosidase per 1 mL of milk. The recombinant BgaP enzyme was added to the milk at 125.7 U_ONPG_/mL. It was mixed with milk at a ratio of 1:9. Sodium phosphate buffer (50 mM, pH 6.6) was added to milk as a negative control. Hydrolysis was halted by the addition of 17 µL of 20% H_2_SO_4_ to 1 mL of the reaction mixture, and the sample was centrifuged (10,000 × *g*, 10 min, 4 °C) and filtered to remove denatured proteins. The quantities of lactose, glucose, and galactose were determined by HPLC using an Aminex HPX-87 H column (Bio-Rad Laboratories, Hercules, CA, USA) incubated at 60 °C with 5 mM H_2_SO_4_ as the mobile phase at a flow rate of 0.6 mL/min, and an Agilent 1200 Series chromatograph with a Refractive Index Detector (Agilent Technologies, Santa Clara, CA, USA).

### Hydrolysis of lactulose

The hydrolysis of lactulose (4.5% in 50 mM sodium phosphate buffer, pH 6.6) by recombinant BgaP was performed at 10 and 30 °C for 24 h. The β-galactosidase was mixed with lactulose in a ratio of 1:9, giving 15.1 U_ONPG_ of β-galactosidase per 1 mL of the reaction mixture. Hydrolysis was halted by the thermal denaturation of BgaP β-galactosidase at 95 °C for 10 min. The sample was then centrifuged and filtered to remove denatured enzyme, and loaded onto an Aminex HPX-87 C column (Bio-Rad Laboratories, Hercules, CA, USA) incubated at 80 °C, with deionized water as the mobile phase at a flow rate of 0.6 mL/min, and an Agilent 1200 Series chromatograph with a Refractive Index Detector (Agilent Technologies, Santa Clara, CA, USA). Lactulose, fructose and galactose were used as standards.

### Hydrolysis of lactose in the presence of l-arabinose isomerase

The hydrolysis of lactose (5.5% in 50 mM sodium phosphate buffer pH 6.6) by the BgaP enzyme in the presence of recombinant l-arabinose isomerase from *Arthrobacter* sp. 22c was performed at 10 and 30 °C for 24 h. Enzymes were mixed with lactose in a ratio of 1:1:8, giving 15.1 U_ONPG_ of β-galactosidase and 0.22 mg (0.05 U_Gal_) of l-arabinose isomerase per 1 mL of the reaction mixture. Samples with a single BgaP or AraA were used as controls. After stopping hydrolysis and isomerization by the heating of samples at 95 °C for 10 min, the sugars were separated by HPLC on an Aminex HPX-87 C column (Bio-Rad Laboratories, Hercules, CA, USA). Lactose, galactose, glucose and tagatose were used as standards. The recombinant *Arthrobacter* sp. 22c AraA enzyme was produced and purified according to Wanarska and Kur^22^.

## Electronic supplementary material

Below is the link to the electronic supplementary material.

Supplementary Material 1

## Data Availability

The DNA sequencing data were deposited in the NCBI database under the accession numbers PQ118618-PQ118623, PQ118646-PQ118654, PQ118663-PQ118679, PQ130458-PQ130471, and PQ130282. The authors declare that the data supporting the findings of this study are available within the article and upon request from the corresponding author.
