# Supplementary material for: Diversity and enzymatic activity of Polish beehive products microbiota, and characterization of a novel β-galactosidase from Paenibacillus sp. 8
Source: Sci Rep. 2025 May 21;15:17625. doi: 10.1038/s41598-025-02561-3 (PMC12095705; doi:10.1038/s41598-025-02561-3)
Supplement: Supplementary file 1 — Supplementary Material 1 [file 41598_2025_2561_MOESM1_ESM.pdf]

Diversity and enzymatic activity of Polish beehive products microbiota, and characterization of a novel β-galactosidase from *Paenibacillus* sp. 8

Aleksandra Rosińska[1], Marta Wanarska[1,*], Katarzyna Kozłowska-Tylingo[2], and Michał Jurkowski[3]

[1]Department of Biotechnology and Microbiology, Faculty of Chemistry, Gdansk University of Technology, Gdansk 80-233, Poland; rosinska.aleks@gmail.com (A. R.)

[2]Department of Pharmaceutical Technology and Biochemistry, Faculty of Chemistry, Gdansk University of Technology, Gdansk 80-233, Poland; katarzyna.kozlowska-tylingo@pg.edu.pl (K. K.-T.)

[3]Department of Physical Chemistry, Faculty of Chemistry, Gdansk University of Technology, Gdansk 80-233, Poland; michal.jurkowski97@gmail.com (M. J.)

*Correspondence: marta.wanarska@pg.edu.pl (M. W.)

Table S1. Identification of selected yeast and fungal isolates based on the D1/D2 region of the large-subunit rDNA and ITS1-5.8S-ITS2 sequences.

Sample	Isolate no.	GenBank accession no.		Closely related strains	D1/D2		ITS	
					GenBank accession no.	Identity (%)	GenBank accession no.	Identity (%)
		D1/D2	ITS					
DH	13	PQ130463	PQ130469	*Starmerella magnoliae* strain CBS 166	NG_060814.1	100.0	KF181964.2	93.65
AH	29	PQ130461	PQ130471	*Starmerella magnoliae* strain CBS 166	NG_060814.1	99.77	KF181964.2	95.61
	30(1)	PQ130462	PQ130467	*Zygosaccharomyces siamensis* strain MinabeTanabe1	LC547999.1	100.0	LC547999.1	99.16
				Zygosaccharomyces siamensis strain ATCC 10685	KC881058.1	100.0	KC881075.1	96.27
HH	61	PQ130460	PQ130466	*Sporobolomyces johnsonii* strain CBS 5470	NG_042343.1	98.61	NR_077090.1	94.24
BH_19	67	PQ130464	PQ130468	*Melnikomyces vietnamensis* strain CBS 136209	NG_058087.1	92.25	NR_166358.1	80.72
				Melnikomyces thailandicus strain CBS 145767	NG_068685.1	91.93	NR_164227.1	80.41
BB	P1	PQ130458	PQ130470	*Starmerella magnoliae* strain CBS 166	NG_060814.1	98.64	KF181964.2	91.21
	P2	PQ130459	PQ130465	*Penicillium chrysogenum* strain CBS 306.48	MH867907.1	99.82	MH856357.1	98.91
				Penicillium tardochrysogenum strain CBS 132200	MH877426.1	99.82	MH865983.1	98.91

AH, acacia honey; BH, buckwheat honey, DH, dandelion honey; HH, honeydew honey; BB, bee bread (perga)

Table S2. Identification of selected bacterial isolates based on the 16S ribosomal RNA gene sequence.

Sample	Isolate no.	GenBank accession no.	Closely related strains	GenBank accession no.	Identity (%)
DH	7	PQ118618	*Bacillus pumilus* strain ATCC 7061	NR_043242.1	99.71
			Bacillus zhangzhouensis strain MCCC 1A08372	NR_148786.1	99.64
	8	PQ118619	*Paenibacillus amylolyticus* strain SQR-21	CP107037.1 (Range: 5465209 to 5466683)	99.93
			Paenibacillus xylanexedens strain PAMC 22703	CP018620.1 (Range: 1482386 to 1483860)	99.93
			Paenibacillus amylolyticus strain NRRL NRS-290	NR_025882.1	99.53
			Paenibacillus xylanexedens strain B22a	NR_044524.1	99.39
			Paenibacillus tundrae strain A10b	NR_044525.1	99.39
	21	PQ118620	*Niallia circulans* strain NBRC 13626	NR_112632.1	99.55
			Niallia circulans strain ATCC 4513	NR_104566.1	99.47
	22	PQ118621	*Metabacillus idriensis* strain SMC 4352-2	NR_043268.1	99.48
	23	PQ118622	*Bacillus pumilus* strain ATCC 7061	NR_043242.1	99.48
			Bacillus zhangzhouensis strain MCCC 1A08372	NR_148786.1	99.41
	33	PQ118623	*Micrococcus aloeverae* strain AE-6	NR_134088.1	99.38
			Micrococcus yunnanensis strain YIM 65004	NR_116578.1	99.22
			Micrococcus luteus strain NCTC 2665	NR_075062.2	98.99
HH	44	PQ118663	*Micrococcus yunnanensis* strain YIM 65004	NR_116578.1	99.08
			Micrococcus aloeverae strain AE-6	NR_134088.1	99.01
			Micrococcus luteus strain NCTC 2665	NR_075062.2	98.93
	51	PQ118664	*Moraxella osloensis* strain DSM 6998	NR_113392.1	99.19
			Moraxella osloensis strain ATCC 19976	NR_113391.1	99.19
	55	PQ118665	*Micrococcus yunnanensis* strain YIM 65004	NR_116578.1	99.16
			Micrococcus luteus strain NCTC 2665	NR_075062.2	99.00
			Micrococcus aloeverae strain AE-6	NR_134088.1	98.93
	59	PQ118666	*Acinetobacter lwoffii* strain DSM 2403	NR_026209.1	99.42
			Acinetobacter lwoffii strain JCM 6840	NR_113346.1	99.42
	63	PQ118667	*Micrococcus yunnanensis* strain YIM 65004	NR_116578.1	99.22
			Micrococcus luteus strain NCTC 2665	NR_075062.2	99.06
			Micrococcus aloeverae strain AE-6	NR_134088.1	98.98
BH_19	77	PQ118668	*Micrococcus yunnanensis* strain YIM 65004	NR_116578.1	99.53
			Micrococcus luteus strain NCTC 2665	NR_075062.2	99.38
			Micrococcus aloeverae strain AE-6	NR_134088.1	99.30
FH	83	PQ118669	*Micrococcus yunnanensis* strain YIM 65004	NR_116578.1	99.16
			Micrococcus luteus strain NCTC 2665	NR_075062.2	98.93
			Micrococcus aloeverae strain AE-6	NR_134088.1	98.85
	90	PQ118670	*Paenibacillus mobilis* strain S8	NR_163642.1	99.55
			Paenibacillus xylanexedens strain B22a	NR_044524.1	99.40
			Paenibacillus amylolyticus strain JCM 9906	NR_112163.1	99.02
BH_23	149	PQ118671	*Micrococcus yunnanensis* strain YIM 65004	NR_116578.1	99.16
			Micrococcus luteus strain NCTC 2665	NR_075062.2	98.93
			Micrococcus aloeverae strain AE-6	NR_134088.1	98.85

HBH	164	PQ118672	*Bacillus wiedmannii* strain FSL W8-0169<br>*Bacillus proteolyticus* strain MCCC 1A00365<br>*Bacillus sanguinis* strain BML-BC004<br>*Bacillus cereus* ATCC 14579	NR_152692.1<br>NR_157735.1<br>NR_175555.1<br>NR_074540.1	99.55<br>99.55<br>99.55<br>99.47
MH_3	172	PQ118673	*Bacillus pumilus* strain ATCC 7061<br>*Bacillus zhangzhouensis* strain MCCC 1A08372	NR_043242.1<br>NR_148786.1	99.34<br>99.27
	176	PQ118674	*Micrococcus yunnanensis* strain YIM 65004<br>*Micrococcus luteus* strain NCTC 2665<br>*Micrococcus aloeverae* strain AE-6	NR_116578.1<br>NR_075062.2<br>NR_134088.1	99.40<br>99.17<br>99.10
MH_49	186	PQ118675	*Micrococcus aloeverae* strain AE-6<br>*Micrococcus yunnanensis* strain YIM 65004<br>*Micrococcus luteus* strain NCTC 2665	NR_134088.1<br>NR_116578.1<br>NR_075062.2	99.39<br>99.16<br>99.00
	187	PQ118676	*Micrococcus yunnanensis* strain YIM 65004<br>*Micrococcus luteus* strain NCTC 2665<br>*Micrococcus aloeverae* strain AE-6	NR_116578.1<br>NR_075062.2<br>NR_134088.1	99.16<br>98.93<br>98.85
	189	PQ118677	*Paenibacillus mobilis* strain S8<br>*Paenibacillus xylanexedens* strain B22a<br>*Paenibacillus amylolyticus* strain NRRL NRS-290	NR_163642.1<br>NR_044524.1<br>NR_025882.1	99.55<br>99.40<br>99.10
	197	PQ118678	*Paenibacillus xylanexedens* strain B22a<br>*Paenibacillus mobilis* strain S8<br>*Paenibacillus amylolyticus* strain NRRL NRS-290	NR_044524.1<br>NR_163642.1<br>NR_025882.1	99.48<br>99.33<br>98.95
BH_97	208	PQ118679	*Micrococcus yunnanensis* strain YIM 65004<br>*Micrococcus luteus* strain NCTC 2665<br>*Micrococcus aloeverae* strain AE-6	NR_116578.1<br>NR_075062.2<br>NR_134088.1	99.24<br>99.01<br>98.94
BB	P3	PQ118646	*Bacillus subtilis* strain DSM 10	NR_027552.1	99.64
	P5	PQ118647	*Micrococcus yunnanensis* strain YIM 65004<br>*Micrococcus luteus* strain NCTC 2665<br>*Micrococcus aloeverae* strain AE-6	NR_116578.1<br>NR_075062.2<br>NR_134088.1	99.47<br>99.32<br>99.24
	P7	PQ118648	*Oceanobacillus profundus* strain CL-MP28	NR_043778.1	99.26
	P8	PQ118649	*Cytobacillus dafuensis* strain FJAT-25496	NR_180561.1	98.69
	P11	PQ118650	*Peribacillus simplex* NBRC 15720 = DSM 1321	NR_112726.1	99.64
	P19	PQ118651	*Paenibacillus xylanexedens* strain B22a<br>*Paenibacillus tundrae* strain A10b<br>*Paenibacillus amylolyticus* strain NRRL NRS-290	NR_044524.1<br>NR_044525.1<br>NR_025882.1	99.66<br>99.12<br>98.98
	P23	PQ118652	*Bacillus pumilus* strain ATCC 7061<br>*Bacillus zhangzhouensis* strain MCCC 1A08372	NR_043242.1<br>NR_148786.1	99.56<br>99.48
	P26	PQ118653	*Micrococcus yunnanensis* strain YIM 65004<br>*Micrococcus luteus* strain NCTC 2665<br>*Micrococcus aloeverae* strain AE-6	NR_116578.1<br>NR_075062.2<br>NR_134088.1	99.27<br>99.05<br>98.98
	P28	PQ118654	*Bacillus mycoides* strain 273	NR_036880.1	99.57

BH, buckwheat honey, DH, dandelion honey; FH, forest honey (multifloral/honeydew); HH, honeydew honey; HBH, honeydew/buckwheat honey; MH, multifloral honey; BB, bee bread (perga)

Table S3. Enzymatic activities of selected bacterial isolates.

Sample	Isolate no.	Enzymatic activity (hydrolyzed substrate)							
		Proteolytic (Casein)	Esterolytic (Tributyrine)	Lipolytic (Tween 20)	Lipolytic (Tween 80)	Amylolytic (Starch)	Cellulolytic (CMC)	Xylanolytic (Xylan)	β-Galactosidase (X-gal)
DH	7	-	++	+	+	-	+	nd	-
	8	+	+	+	-	++	++	+	+++
	21	+	-	+	++	-	-	nd	++
	22	+	-	-	-	++	-	nd	-
	23	++	++	+	-	-	+	nd	-
	33	++	-	++	++	-	-	nd	-
HH	44	++	+	+	+	-	-	nd	-
	51	-	+	+++	+	-	-	nd	-
	55	+++	-	-	-	-	-	nd	-
	59	-	+	+++	++	-	-	nd	-
	63	+++	-	+	++	-	-	nd	-
BH_19	77	+++	-	-	-	-	-	nd	-
FH	83	++	++	++	+	-	-	nd	-
	90	+	+	++	-	+++	++	nd	-
BH_23	149	++	+	++	++	-	-	nd	-
HBH	164	+	-	-	-	++	-	nd	-
MH_3	172	+++	++	-	-	-	+	nd	-
	176	+++	-	-	-	-	-	nd	-
MH_49	186	+++	-	-	-	w	-	nd	-
	187	+	++	+++	++	-	-	nd	-
	189	++	-	-	-	+++	++	nd	w
	197	-	+++	-	-	+++	++	nd	w
BH_97	208	+++	+++	++	+	-	-	nd	-
BB	P3	++	++	++	++	+++	++	-	-
	P5	++	+	-	-	-	-	-	-
	P7	-	-	-	-	-	+	-	-
	P8	-	-	-	+	-	-	-	+
	P11	-	-	+++	+	-	-	-	-
	P19	+	+	++	-	+++	++	+	++
	P23	++	++	-	-	-	++	-	-
	P26	+++	+	+	-	-	-	-	-
	P28	-	+	+	-	-	-	-	-
Symbol	w	nd	nd	nd	nd	HI = 1.1-1.2	nd	nd	Pale blue
	+	HI = 1.8-2.3	HI = 1.2-1.4	HI = 1.2-1.6	HI = 1.2-1.6	HI = 1.6-2.1	HI = 1.7-2.3	HI = 2.2-2.6 (xylans from corncobs and beech wood)	Light blue
	++	HI = 2.4-2.9	HI = 1.5-1.7	HI = 1.7-2.1	HI = 1.7-2.1	HI = 2.2-2.6	HI = 2.4-2.7		Blue
	+++	HI = 3.0-3.5	HI = 1.8-2.0	HI = 2.2-2.6	HI = 2.2-2.6	HI = 2.7-3.2	HI = 2.8-3.3		Dark blue

BH, buckwheat honey; DH, dandelion honey; FH, forest honey (multifloral/honeydew); HH, honeydew honey; HBH, honeydew/buckwheat honey; MH, multifloral honey; BB, bee bread (perga); CMC, carboxymethylcellulose; X-gal, 5-bromo-4-chloro-3-indolyl-β-D-galactopyranoside; (-) negative; (w) weakly positive; (+) positive; (++) moderately positive; (+++) strongly positive; HI (Halo Zone Index) = Halo zone diameter with colony diameter/Colony diameter; nd, not determined

Table S4. Substrate specificity of the recombinant *Paenibacillus* sp. 8 β-galactosidase.

**Substrate**	**Relative activity (%)**
p-Nitrophenyl-β-D-galactopyranoside	100
p-Nitrophenyl-α-D-galactopyranoside	< 0.1
p-Nitrophenyl-β-D-glucopyranoside	< 0.1
p-Nitrophenyl-α-D-glucopyranoside	< 0.1
p-Nitrophenyl-β-D-fucopyranoside	< 0.1
p-Nitrophenyl-β-D-xylopyranoside	< 0.1
p-Nitrophenyl-β-L-arabinofuranoside	< 0.1
p-Nitrophenyl-β-D-glucuronide	< 0.1
p-Nitrophenyl-β-D-mannopyranoside	< 0.1
p-Nitrophenyl-β-D-cellobioside	< 0.1

```
Paenibacillus      MISNKLPKMFYGGDYNPEQWDHET-HLEDLRMFQLAGIDIATINVFSWALIQPDEVTYRF   59
Planococcus        MINDKLPKIWHGGDYNPEQWDSQEIWDEDVRMFKLAGIDVATLNVFSWALNQPNEDTYNF   60
Niallia            MINEKFPKIWYGGDYNPEQWDKAT-MEEDMRMFNLAGIDVATVNVFSWAKIQRDEVSYDF   59
                   **.:*:**:::**********      **:***:*****:**:******  * :* :* *

Paenibacillus      EELDQLINRLHENGVYICLATSTAAHPAWMAKKYPDVLRVDADGRKRKFGGRHNSCPNSP   119
Planococcus        EWLDDKINRLYENGIYTCLATSTAAHPAWMAKKYPDVLRVDFYGRKRKFGSRHNSCPNSP   120
Niallia            TWLDDIIERLTKENIYLCLATSTGAHPAWMAKKYPDVLRVDYEGRKRKFGGRHNSCPNSP   119
                     **: *:** ::.:* ******.*****************  *******.*********

Paenibacillus      TYRKYAEKIADKLAERYKDHPAVLVWHISNEYGGECYCDNCEKAFRVYLKERYQTLEQVN   179
Planococcus        TYREYSEKIADKLAERYKDHPAVLIWHVSNEYGGYCYCDNCQDAFRVWLSDKYGTLEKLN   180
Niallia            TYRKYAKILAGKLAERYKDHPQIVMWHVSNEYGGYCYCDNCEKQFRVWLKERYGTLEALN   179
                   ***:*:: :*.********** :::**:****** ******:. ***:*.::* *** :*

Paenibacillus      KAWNTNFWGHTFYDWDEIVLPSNLSEHWGNNNSTFQGISLDYSRFNSDSMLDCYLLEYDA   239
Planococcus        KAWNTGFWGHTFYEWDEIVAPNMLSEEREDNVSDFQGISLDYRRFQSDSLLDCYKLEYNA   240
Niallia            KAWNTSFWSHTFYDWDEIVAPNALSEEWSGNRTNFQGISLDYRRFQSDSLLECFKMERDE   239
                   *****.**.****:***** *. ***.  .* : ******** **:***:*:*: :* :

Paenibacillus      IKKHIPDSVVTTNLMGFFKQLDYFKWAKYLDIVSWDSYPGLATPVSFTAMAHDLMRGLKD   299
Planococcus        IRKHTPNIPITTNLMGTYPMLDYFKWAKEMDVVSWDNYPAIDTPFSYTAMTHDLMRGLKS   300
Niallia            LKRWTPDIPVTTNLMGFYPELDYFKWAKEMDVVSWDNYPSMDTPFSFTAMAHNLMRGLKS   299
                   :::  *:  :****** :  ******** :*:****.**.: **.*:***:*:******.

Paenibacillus      GQPFMLMEQTPSQQNWQPYNSLKRPGVMRLWSYQSVAHGADTIMFFQLRRSIGACEKYHG   359
Planococcus        GQPFMLMEQTPSQQNWQPYNSLKRPGVMRLWSYQAIGRGADTILYFQLRRSVGACEKYHG   360
Niallia            GQPFMLMEQTPGVQNWQPYNSAKRPGVMRLWSYQAVAHGADTVMFFQLRRSVGACEKYHG   359
                   ***********. ******** ************::.:****:::******:********

Paenibacillus      AVIEHAGHENTRVFREVAELGKELQILGDTTLDASVESKVAIVFDWDNWWAIEKSSGPTV   419
Planococcus        AVIEHVGHEHTRVFNEVAQIGKEFNQLGDTLLDARVNARVAIVFDWENRWATELSSGPSV   420
Niallia            AVIEHVGHEHTRVFRECAELGKELQQLGDTILDARSEAKVAVMYDWENRWALELSSGPSI   419
                   *****.***:****.* *::***:: **** ***  :::**:::**:* ** * ****::

Paenibacillus      ALNYVDQIHKYYAAFFRRNIQVDIISVDTDMSKYDIVLAPVLYMVKPGFAAKLEKYVEAG   479
Planococcus        SLDYVNEVHKYYDALYKLNVQVDMVGVEEDLSQYDVVIAPVLYMVKEGYAAKVESFVENG   480
Niallia            ALNYVNEVHKYYDALYKQNIQTDMISVEEDLSKYKVVIAPVMYMVKPGFAERVERFVAQG   479
                   :*:**:::**** *::: *:*.*::.*: *:*:*.:*:***:**** *:* ::* :*  *

Paenibacillus      GTFLTTFFSGIVNENDLVTTGGYPGELRKLLGIWVEEIDALLPEQKNRIVLKETYGDLEG   539
Planococcus        GTFITTFFSGIVNETDIVTLGGYPGELRKVLGIWAEEIDALHPDETNEIVVNGSRGSLSG   540
Niallia            GTFVTTFFSGIVNENDLVTLGGYPGELRNVMGIWAEEIDALLPGHQNEIVLRQDWGGLRG   539
                   ***:**********.*:** ********:::***.****** * . *.**:.   *.* *

Paenibacillus      EYGCGMLCDLLHSEGAEVIAEYGDDFYKGMPVVTRNTFGQGEAWYVASDPDERFLDGLLG   599
Planococcus        SYSCNLLFDLIHTEGAQAVAEYGSDFYQGMPVLTVNEFGKGKAWYVASSPDAEFLVDFLQ   600
Niallia            SYSCGILCDVIHAETAEVLAEYGADYYKGTPVLTRNKFGNGQSYYVASSPDADFLQGLIA   599
                   .*.*.:* *::*:* *:.:**** *:*:* **:* * **:*:::****.**  ** .::

Paenibacillus      QLAAAKNIESLLETPEGVEVSARTKDGKPYLFVMNHNATTQSYDLGTAKAHDLLTNRELS   659
Planococcus        TVCEEAGVEPLLSVPEGVETTERVKDGQTYLFVLNHNNKVESIDLKDSQYQELLSTQQLS   660
Niallia            NLCEEQGVKPLLNTPDGVEVAERVKNGTSYLFVMNHNAEEMTFDAGASRQRDLLTGKTIS   659
                    :.   .:: **..*:***.: *.*:*  ****:***    : *   :: ::**: : :*

Paenibacillus      GSVEIEARGVQLLEMK   675
Planococcus        GTVELEAKGVFILAKV   676
Niallia            GQATIPARGVMILERA   675
                   * . : *:** :*
```

Fig. S1. The multiple sequence alignment of *Paenibacillus* sp. 8 BgaP enzyme (GenBank, accession no. XED97098) with β-galactosidases from mesophilic *Niallia circulans* subsp. *alkalophilus* (RCSB PDB, accession no. 3TTS) and psychrotolerant *Planococcus* sp. L4 (GenBank, accession no. ABI64125). Catalytic residues E150 and E307 are shaded red, cysteine residues of the metal binding site are shaded yellow, and the amino acid residues involved in the α-galactose binding are shaded green. The multiple sequence alignment was performed using Clustal Omega (1.2.4) program (https://www.ebi.ac.uk/jdispatcher/msa/clustalo).

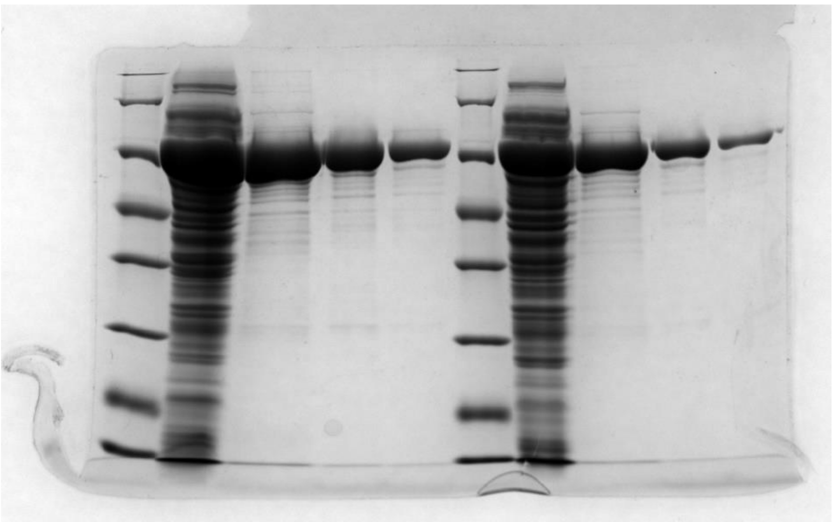

Fig. 2. Original SDS-PAGE gel.
